# Full spectrum of vitamin D immunomodulation in multiple sclerosis: mechanisms and therapeutic implications

**DOI:** 10.1093/braincomms/fcac171

**Published:** 2022-06-30

**Authors:** Manon Galoppin, Saniya Kari, Sasha Soldati, Arindam Pal, Manon Rival, Britta Engelhardt, Anne Astier, Eric Thouvenot

**Affiliations:** IGF, University Montpellier, CNRS, INSERM, Montpellier, France; Toulouse Institute for Infectious and Inflammatory Diseases (Infinity), INSERM UMR1291 – CNRS UMR5051 – Université Toulouse III, 31024 Toulouse cedex 3, France; Theodor Kocher Institute, University of Bern, Bern, Switzerland; Theodor Kocher Institute, University of Bern, Bern, Switzerland; IGF, University Montpellier, CNRS, INSERM, Montpellier, France; Department of Neurology, Nîmes University Hospital, University Montpellier, Nîmes, France; Theodor Kocher Institute, University of Bern, Bern, Switzerland; Toulouse Institute for Infectious and Inflammatory Diseases (Infinity), INSERM UMR1291 – CNRS UMR5051 – Université Toulouse III, 31024 Toulouse cedex 3, France; IGF, University Montpellier, CNRS, INSERM, Montpellier, France; Department of Neurology, Nîmes University Hospital, University Montpellier, Nîmes, France

**Keywords:** vitamin D, multiple sclerosis, cytokines, lymphocytes, immunomodulation

## Abstract

Vitamin D deficiency has been associated with the risk of multiple sclerosis, disease activity and progression. Results from *in vitro* experiments, animal models and analysis of human samples from randomized controlled trials provide comprehensive data illustrating the pleiotropic actions of Vitamin D on the immune system. They globally result in immunomodulation by decreasing differentiation of effector T and B cells while promoting regulatory subsets. Vitamin D also modulates innate immune cells such as macrophages, monocytes and dendritic cells, and acts at the level of the blood–brain barrier reducing immune cell trafficking. Vitamin D exerts additional activity within the central nervous system reducing microglial and astrocytic activation. The immunomodulatory role of Vitamin D detected in animal models of multiple sclerosis has suggested its potential therapeutic use for treating multiple sclerosis. In this review, we focus on recent published data describing the biological effects of Vitamin D in animal models of multiple sclerosis on immune cells, blood–brain barrier function, activation of glial cells and its potential neuroprotective effects. Based on the current knowledge, we also discuss optimization of therapeutic interventions with Vitamin D in patients with multiple sclerosis, as well as new technologies allowing in-depth analysis of immune cell regulations by vitamin D.

## Teaser

Galoppin *et al*. provide a comprehensive analysis of the biological effects of Vitamin D (VitD) in animal models and patients with multiple sclerosis, especially focusing on regulation of immune cells, blood–brain barrier function, activation of glial cells and therapeutic trials. They discuss optimization of therapeutic interventions with VitD in multiple sclerosis.

## Graphical Abstract

**Graphical Abstract:**
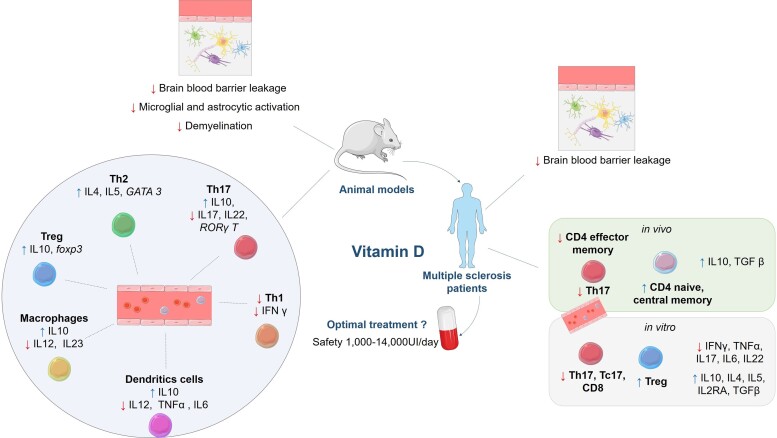

## Introduction

Multiple sclerosis is a chronic inflammatory and neurodegenerative disease of the CNS especially affecting women (3:1 sex ratio). Eighty-five per cent of patients develop multiple sclerosis with a clinically isolated syndrome corresponding to an acute demyelination of the optic nerves, brainstem or spinal cord, followed by recovery. Most multiple sclerosis patients experience subsequent relapses (relapsing-remitting multiple sclerosis—RRMS) with some later converting to secondary progressive multiple sclerosis (SPMS), characterized by gradual neurological deterioration, with or without superimposed relapses. In 15% of cases, neurological symptoms progress from onset (primary progressive multiple sclerosis), with equal frequency in males and females.

Epidemiological studies have highlighted a familial aggregation of multiple sclerosis potentially explained by several environmental and genetic risk factors.^1^ Family studies assessing risks to relatives of multiple sclerosis patients showed that first-degree relatives are generally at 10–25 times greater risk of developing multiple sclerosis than the general population, but this risk could be lower.^2^ Several major histocompatibility complex (MHC) loci—especially HLA DRB1*15:01—are the most important genetic risk factors for developing multiple sclerosis with odd ratios around three. Moreover, genome wide association studies (GWAS) studies have identified more than 200 susceptibility variants other than MHC that implicate multiple innate and adaptive immune pathways distributed across the cellular components of the immune system and brain-resident immune cells, highlighting the importance of altered peripheral immune cells and glial cells responses in multiple sclerosis pathophysiology.^3^

The main environmental risk factors for multiple sclerosis include smoking,^4,5^ obesity,^6^ Epstein-Barr Virus infection,^7,8^ sun exposure^9,10^ and vitamin D (VitD) deficiency.^11^ The latter has been recognized as a major risk factor for multiple sclerosis, with circulating levels modulated by specific genetic variants, skin colour and sun exposure, especially in early years of life.^2^ Many reports have highlighted the clinical correlation of circulating VitD levels in multiple sclerosis with clinical disease activity^12,13^ and of disability progression.^9,14^ Moreover, VitD concentrations are significantly lower in females compared with males^15^ and lack of sunlight and VitD deficiency seem to further increase the risk of multiple sclerosis in females than in males.^16^

**Figure 1:**
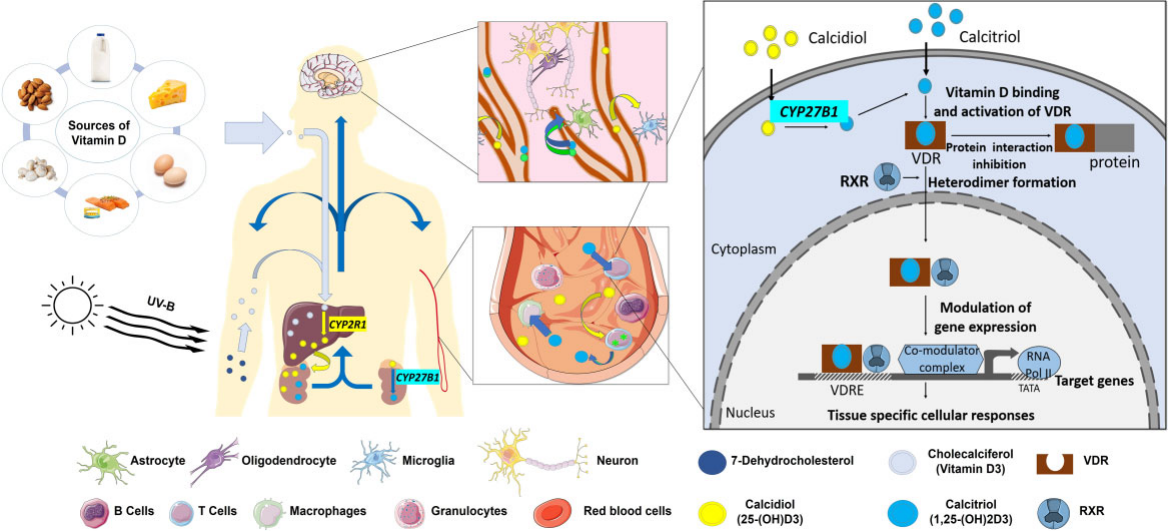
**Sources, metabolism and cellular mode of action of VitD.** VitD is synthesized in the skin under the effect of UV radiation and is also provided by food sources. Cholecalciferol (inactive form) is stored in adipose tissue and transformed in the liver into 25-hydroxyvitamin D_3_ [25(OH)D_3_, calcidiol—main circulating form]. Calcidiol is then hydroxylated in the kidney to form 1,25-dihydroxyvitamin D_3_ [1,25(OH)_2_D_3_, calcitriol—active metabolite]. VitD metabolites circulate ubiquitously, bound to its transporter: Vit DBP and is able to cross the blood–brain barrier to modulate CNS-resident cells (neurons, astrocytes, oligodendrocytes and microglia). Calcitriol acts intracellularly as a transcription factor by binding to the cytoplasmic VDR which heterodimerizes with RXR. Upon translocation of this heterodimer to the nucleus, VitD/VDR/RXR complex binds to the VDRE in the genomic DNA and modulates the expression of target genes. Drawings of the individual cell types were adapted from Servier Medical Art (http://smart.servier.com/), licenced under a Creative Common Attribution 3.0 Generic License.

There are different sources of VitD. Cholecalciferol (VitD_3_) is a lipophilic cholesterol derivative (Fig. 1) synthesized in the skin from 7-dehydrocholesterol under the effect of UV radiation. It is also provided by animal food sources, especially oily fish. Cholecalciferol, which is biologically inactive, is stored in adipose tissue and transformed in the liver into 25-hydroxyvitamin D_3_ [25(OH)D_3_, calcidiol], which is the main circulating form of VitD. Calcidiol is then hydroxylated in the kidney to form 1,25-dihydroxyvitamin D_3_ [1,25(OH)_2_D_3_, calcitriol], which is the active metabolite of VitD.^17^ VitD metabolites circulate ubiquitously, bound to VitD binding protein (DBP), its transporter. Calcitriol acts intracellularly as a transcription factor by binding to the VitD receptor (VDR), which heterodimerizes with retinoid-X-receptor (RXR).^18,19^ Upon translocation of this heterodimer to the nucleus, VitD recognizes VitD responsive elements (VDREs) in the genomic DNA and modulates the expression of over 200 genes. Most of these are associated with immune functions and VitD metabolism, highlighting the key role of VitD deficiency in numerous inflammatory diseases and cancer.^1^

The pleiotropic actions of VitD on the immune system globally result in immunomodulation by decreasing differentiation of effector T and B cells while promoting regulatory subsets.^20^ VitD is also known to modulate cells of the innate immune system such as macrophages, monocytes and dendritic cells (DCs), that additionally contribute to multiple sclerosis pathophysiology.^20^ Furthermore, VitD acts in the CNS by regulating microglial and astrocytic activation.^21^ The immunomodulatory role of VitD in animal models of multiple sclerosis has suggested its potential therapeutic use in multiple sclerosis. In this context, a comprehensive analysis of data concerning the *in vitro* and *in vivo* effects of VitD in multiple sclerosis is mandatory to optimize its use in clinical practice. In this review, we focus on recent published data describing the biological effects of VitD in animal models of multiple sclerosis, on immune cells, blood–brain barrier (BBB) function, activation of glial cells and its potential neuroprotective effects. Based on the current knowledge, we discuss the optimization of therapeutic interventions with VitD in multiple sclerosis patients.

## Immune cells and animal models

### VitD ameliorates experimental autoimmune encephalomyelitis

Experimental autoimmune encephalitis (EAE), the main animal model of multiple sclerosis, is based on immunization of susceptible animals (especially rodents) with myelin antigens to trigger an autoinflammatory response in the CNS. Used in multiple strains or genotypes of rodents, it has been largely developed to recapitulate different aspects of the disease (clinical course, lesion localization or pathological patterns) and has significantly contributed to our understanding of the immunopathologic features of multiple sclerosis.^22^

EAE can be actively induced (active EAE) by the immunization of animals with peptides in emulsion of incomplete Freund’s adjuvant containing heat killed Mycobacterium tuberculosis (H37Ra) in combination with pertussis toxin. Peptide injection triggers activation of myelin antigen-specific T cells and leads to CNS autoimmunity.^23^ Alternatively, passive EAE is induced by the adoptive transfer of pre-activated myelin-specific T cells into naïve syngeneic recipients. The development of clinical EAE can be quantified using different clinical EAE scoring systems.^23,24^

Most used EAE models include immunization with peptides from myelin oligodendrocyte glycoprotein (MOG) (e.g. MOG_35−55_) in C57BL/6 mice,^25–30^ with myelin basic protein (MBP) in B10.PL mice or in Lewis rats^29,31–35^ or with proteolipid protein (PLP) (e.g. PLP_139–151_) in SJL mice.^27,28,36^

The efficacy of calcitriol in active EAE models has been analysed in a prophylactic or curative approach. Most studies show a clear protective effect of VitD in EAE. In the prophylactic method, a supplementation of calcitriol is added to the food before induction of EAE (generally, 50 ng/day for females and 200 ng/day for males).^32^ In the curative method, calcitriol is administrated after the onset of clinical signs by intraperitoneal injection (50–300 ng), which may be combined with a supplementation in food (50 ng/day). Prophylactic administration of calcitriol inhibited EAE development, and therapeutic administration decreased the severity of active and passive EAE in B10.PL, C57BL/6 and SJL/J.^25–27,31,32,34,36,37^ Moreover, cholecalciferol also reduced EAE severity in prevention.^29,33,35^ In contrast, cholecalciferol alone failed to reverse the disease in the therapeutic setting in the MBP B10.PL mouse EAE model, while the combination of cholecalciferol and calcitriol did.^38^

An interesting study showed that the protective effect of VitD in EAE is transient as removal of VitD resulted in an increased severity of the disease in MOG-induced EAE in C57BL/6 mice.^26^ This study also suggested that calcitriol does not inhibit the production of autoreactive CD4^+^ T cells but that it may block their migration to the CNS. Indeed, there are fewer mononuclear cells infiltrating the CNS tissue after the induction of active EAE in mice and rats treated with cholecalciferol or calcitriol.^25,27,33,34,37^ Calcitriol may decrease the migration of encephalitogenic T cells via the regulation of adhesion molecules involved in their trafficking into the CNS, as suggested by the lowered CXCR3 expression on CD4^+^ T cells in calcitriol-treated mice (MOG-induced EAE in the C57BL/6 model).^26^  *In vitro*, calcitriol reduced CCR6 expression and migration of Th17 cells.^25^ Furthermore, reduced secretion of pro-inflammatory interleukin (IL)-17A and IL-22 and increased production of anti-inflammatory IL-10 by Foxp3^+^ CD4^+^ CD25^high^ regulatory T cells (Tregs) was detected in the brain of PLP_139–151_ EAE SJL/J mice treated with calcitriol.^27^

Several important studies have demonstrated the key role of the VDR in mediating the protective effect of VitD in EAE. VDR knockout (VDR KO) C57BL/6 mice with deleted exons coding for the first or second zinc finger of the DNA binding domain (VDR^tm1Ska^-KO and VDR^tm1Mbd^-KO, respectively) have been used.^39,40^ In the absence of VDR, calcitriol does not confer a protection to active EAE in C57BL/6 VDR^tm1Ska^-KO mice.^41^ Furthermore, calcitriol failed to inhibit EAE in C57BL/6 mice lacking functional VDR specifically in CD4^+^ T lymphocytes,^41^ demonstrating that VDR expression in CD4^+^ T cells is key for its protective effect.^42^ Moreover, both *in vitro* and *in vivo*, an increase in autoreactive Th17 responses and a decrease in tolerogenic CD103^+^ CD11c^+^ DCs were observed in VDR KO mice.^43^ Taken together, VDR seems indispensable to mediate the anti-inflammatory responses mediated by VitD.

### Circulating lymphocytes

#### CD4^+^ Th1 and Th17 cells

**Figure 2:**
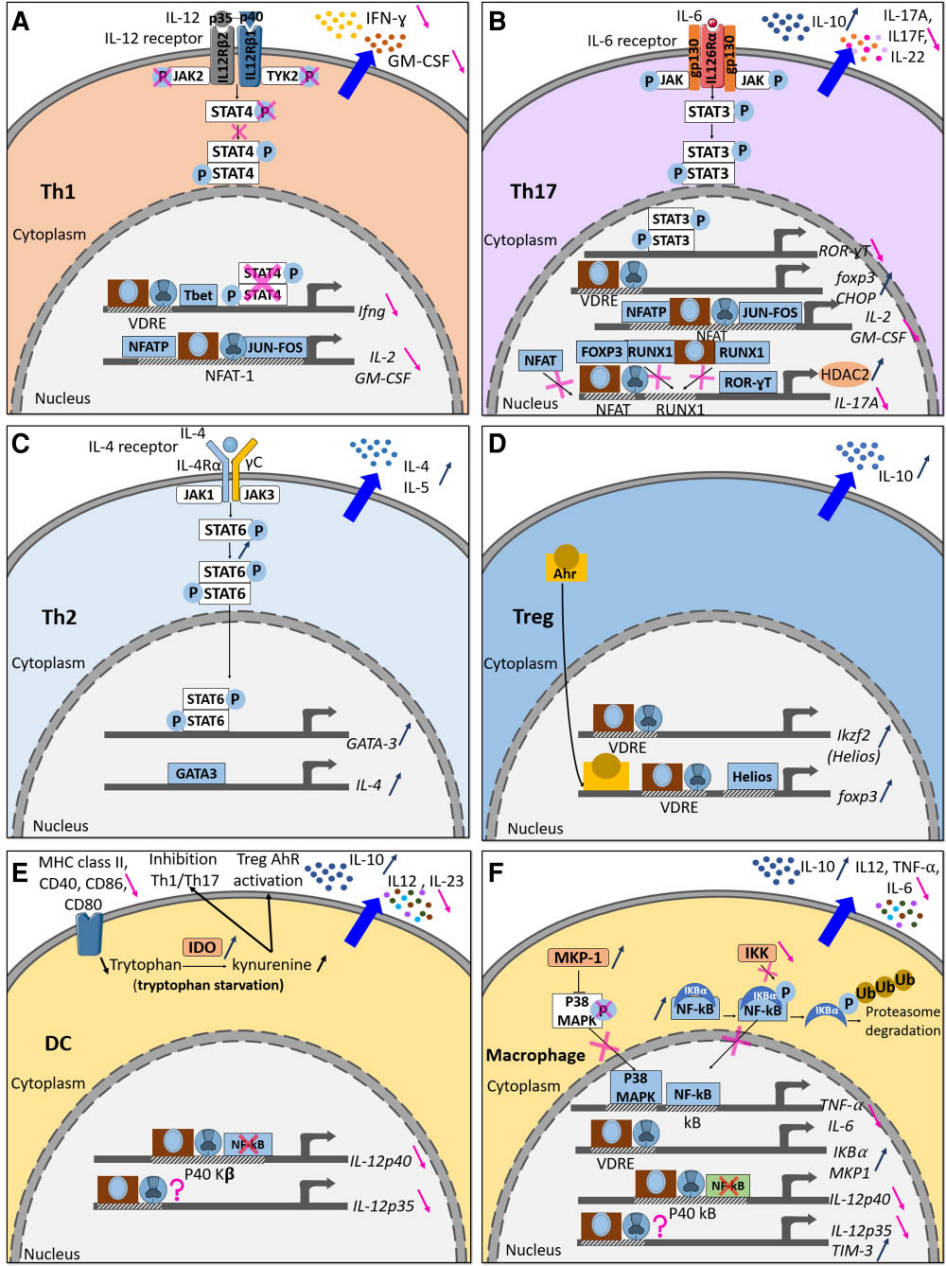
**Immunomodulatory effects of VitD in mice.** VitD has both a direct and indirect immunosuppressive effect on Th1 (**A**), Th17 (**B**), Th2 (**C**), Treg (**D**), DC (**E**) and macrophage (**F**).

Most immunomodulatory effects of VitD have been well described in CD4+ T lymphocytes. Calcitriol exerts strong and direct immunomodulatory effects when added to CD4^+^ T-cell cultures *in vitro*, by inhibiting effector Th1 and Th17 and promoting Th2 and Tregs calcitriol inhibits the differentiation of naïve CD62L^+^CD4^+^ T cells into Th1 and Th17 cells^25,44^ and reduces the production of interferon gamma (IFN-γ) by Th1 cells.^45–47^ In Jurkat T cells, the VDR/RXR complex was found to bind to a silencer region in the human IFN-γ promoter^46^ (Fig. 2A). However, it is important to note that the decreased IFN-γ production could also result from an indirect action of calcitriol on IL-12 production by DCs and macrophages,^28,48^ as further described below, as IL-12 promotes IFN-γ production and Th1 differentiation via the activation of STAT4^49^ (Fig. 2E). *In vitro* treatment of activated T cells from SJL/J mice with calcitriol inhibits IL-12-induced tyrosine phosphorylation of STAT3, STAT4 and of the kinases JAK2 and TYK2^28^ (Fig. 2A). Therefore, the modulation of the JAK-STAT signalling pathway by VitD impairs the development of Th1 cells.

Several studies have shown that calcitriol also inhibits IL-17A, IL-22 and IL-17F production by Th17 cells from EAE mice, both *in vivo* and *in vitro*.^27,50,51^ Calcitriol reduces the level of mRNA that encodes IL-17A, IL-17F and IL-22, suggesting that inhibition of cytokines in Th17 cells occurs via transcriptional modulation^51^ (Fig. 2B). The nuclear factor of activated T cells (NFAT)-AP-1 complex is important for the induction of IL-17 and IL-2 expression in CD4^+^ T cells.^52^ In one study using HUT102 cells and Jurkat T cells treated with calcitriol, there is a competition of calcitriol–VDR–RXR complex and NFAT to NFAT-binding sites in the gene coding IL-17A. However, in mice, the repression of IL-17A is independent of NFAT (Fig. 2B). Furthermore, in both human cell lines and in mice, binding of the calcitriol–VDR–RXR complex leads to the transcriptional repression of IL-17A expression through the recruitment of Histone Deacetylase 2.^27^ In a similar fashion, IL-2 and GM-CSF are inhibited.^53,54^ The VDR–RXR complex interferes with the formation of the NFATp–Jun–Fos transcriptional complex on the promoter of the IL-2 gene. RUNX1 has a key role in the differentiation of Th17 cells and the transcription of IL-17A. VDR binds to RUNX1 and prevents its binding to the IL-17a gene promoter. In addition, there is the formation of an inhibitory complex between FOXP3 and RUNX1, which blocks the activation of IL-17A (Fig. 2B). However, Chang *et al*. found no significance difference of the level of IL-17A mRNA upon addition of calcitriol in MOG_35–55_ EAE in C57BL/6 mice. They proposed that the reduction of production of cytokines results from a translational regulation. *In vitro*, C/EBP homologous protein, endoplasmic reticulum stress sensor is induced by calcitriol in Th17 cells.^50^

#### CD4^+^ Th2 cells

In contrast to Th1 and Th17, several studies have shown that calcitriol favours Th2 differentiation.^30,37,44^ Following the addition of calcitriol to CD4+ T cells from BALB/C and C57BL/6 mice, a significant increase in *GATA-3*, a Th2-specific transcription factor, and c-maf was observed (Fig. 2C). Indeed, the Th2 signature cytokines IL-4 and IL-5 were upregulated by calcitriol while expression of IFN-γ and IL-17 was decreased. Moreover, STAT6 is important for calcitriol to confer a protection of MOG_35–55_ EAE in CD57BL/6 mice (Fig. 2C).

#### Regulatory T cells

Tregs play an important role in maintaining peripheral tolerance.^55^ Impaired Foxp3^+^ CD4^+^ CD25^high^ Treg have been identified as a key feature of multiple sclerosis.^56^ Many studies have highlighted the role of VitD in promoting Tregs, and of the secretion of anti-inflammatory IL-10. Calcitriol and cholecalciferol increased the numbers of Tregs in the periphery and in the spinal cord of MOG_35–55_ induced EAE in C57BL/6 mice and MBP-induced EAE in B10.PL mice and Lewis rats.^27,33,37,38^ Moreover, calcitriol increased their production of IL-10^57^ (Fig. 2D). Indeed, key studies have shown that a functional IL-10 pathway is necessary for 1,25(OH)_2_D_3_ to inhibit EAE.^51,58,59^ Few studies have also reported an important role of VitD in Foxp3 regulation. The increase in Tregs is due to increased transcription of the *foxp3* gene as VDRE are present in the murine Foxp3 promoter.^27^ Calcitriol also indirectly increased FOXP3 expression through the modulation of Helios, a positive regulator of FOXP3, and a marker of thymic-derived natural Tregs.^60^ Injection of calcitriol (200 ng in oil) or the combination of calcitriol and cholecalciferol (gavage 5 μg in oil) increased Helios^+^Foxp3^+^ Treg in MOG_35–55_ EAE in C57BL/6 mice and MBP EAE in B10.PL mice.^38,61^ VitD increases the expression of *Ikzf2* (encoding Helios) in CD4^+^ T cells in a VDR-dependent mechanism,^61^ and Helios was shown to bind to the *foxp3* promoter and increase its expression, like the aryl-hydrocarbon receptor (Ahr) does^62,63^ (Fig. 2D).

In addition, there is evidence that VitD signalling plays a greater role in maintaining T-cell tolerance in females than in males. In this interesting study, a cooperative loop between VitD and oestrogen has been described as calcitriol promotes oestradiol (E2) that reduces transcription of *Cyp24a1*, the catabolic enzyme for active vitamin, and increases VDR in T cells, which in turn, promotes Treg development.^29,64^ Therefore, lowered VitD and sex-specific VitD signalling may contribute to the breakdown in T-cell self-tolerance and increased multiple sclerosis incidence in females.

#### B cells

Much of the current literature has focused on CD4^+^ T cells. However, understanding the role of VitD on B cells is essential, given the high efficacy of anti-CD20 treatments in multiple sclerosis.^65^ VitD has been shown to modulate human B cells, by regulating their proliferation, cytokine secretion and differentiation into plasma cells.^20^ However, as far as we know, no study analysed the direct role of VitD on B cells in EAE. One study reported the indirect effect of calcitriol through tolerogenic DCs in B cells.^66^ The transfer of tolerogenic DCs induced by calcitriol in MOG_35–55_ EAE in C57BL/6 mice increased the proportion of CD19^+^ CD5^+^ CD1d^+^ regulatory B cells (Breg) in the spleen. Breg play an important role in MOG_G35–55_ EAE C57BL/6 mice by increasing the production of IL-10.^67^

### Innate immune cells

#### Dendritic cells

DCs are antigen presenting cells (APCs) that participate in the initiation and development of multiple sclerosis.^68^ In the periphery, DCs activate naïve CD4^+^ T cells into CD4^+^ Th1 and Th17 cells, that in turn cross the BBB and are reactivated in the perivascular and subarachnoid CNS spaces by resident APCs allowing them to infiltrate the CNS parenchyma.^69,70^

Besides the established role of VitD in T cells, a clear role in DCs has also been clearly demonstrated. Addition of calcitriol generates tolerogenic DCs. Most studies have generated tolerogenic DCs *in vitro* and transferred them to MOG_35–55_ EAE in C57BL/6 or MBP_73–86_ EAE in Lewis rats.^66,71^ Tolerogenic DCs are generated by adding GM-CSF (10 ng/ml) and/or IL-4 (10 ng/ml) and calcitriol (1–10 nM) or cholecalciferol (1 nM) to progenitor bone marrow cells from female CD57BL/6 mice or Lewis rats. They are characterized by a decreased expression of MHC Class II, CD40, CD86 and CD80 (co-stimulatory molecules), decreased IL-12, IL-23 and increased IL-10 production compared with untreated DCs^66,71,72^ (Fig. 2E). DCs are generally pulsed with MOG_35–55_ peptide before transfer in EAE models at different time points: before immunization (preventive), after immunization (preclinical) and after the onset of the disease (therapeutic). This approach allows to determine the direct impact of VitD on DCs and its indirect effect on the adaptive immune response. The administration of tolerogenic DCs after immunization and after the onset of the disease led to a reduction of the severity of the disease in MOG_35–55_ EAE in C57BL/6 mice.^66,71^ In the preventive approach, tolerogenic DCs abrogated EAE in MOG_35–55_ EAE in C57BL/6 mice and Lewis rat.^33,71^ Furthermore, treatment of EAE by tolerogenic DCs led to an increase in Tregs and Bregs and secretion of IL-10,^33,66,71^ with a reduced infiltration of autoreactive Th1 and Th17 cells into the spinal cord^66^ and in a reduction of CD11c^+^ DCs^66,73^ (Fig. 2E).

Mechanistically, cholecalciferol and calcitriol increased the expression of indoleamine 2,3-dioxygenase (IDO), an enzyme that converts tryptophan to kynurenine (Fig. 2E). The inhibition of IDO on tolerogenic DCs abrogated the effect of cholecalciferol on MBP_73–86_ EAE course in Lewis rats.^33^ IDO^+^-tolerogenic DCs led to the generation of Treg through two mechanisms: tryptophan deficiency^74^ and the activation of AhR on CD4^+^ T cells by kynurenine, first metabolite of IDO.^63,75^

As mentioned earlier, it is also demonstrated that VitD reduces IL-12 production by myeloid cells. It inhibits IL-12p35 and p40 subunits at the transcriptional level in DCs and macrophages (Fig. 2E and F). Furthermore, calcitriol inhibits the mRNA expression of the IL-12p40 subunit in a VDR-dependent manner. Calcitriol may interfere with the binding of nuclear factor-κB (NF-kB) on the NF-kB binding site in the p40 promoter by competition with VDR–RXR heterodimers^48^ or by downregulation of the activation of the NF-kB signalling pathway in DCs and macrophages.^76^

#### Monocytes/macrophages

Similarly to DCs, VitD modulates their function. In macrophages, calcitriol inhibits the production of tumour necrosis factor-α (TNFα) by reducing the activity of a major transcription factor (NF-kB), through the increase of inhibitor of kB-α (IkB-α) that sequesters NF-kB in the cytoplasm of these cells (Fig. 2F). The phosphorylation of IkB-α by IkB kinase (IKK) leads to its ubiquitination and degradation by the proteasome. It allows the release of NF-kB that can translocate to the nucleus where it binds to kB site on TNFα promoter and activates its expression. Calcitriol binds to the promoter of IkB-α and upregulates its mRNA expression, leading to an increase of IkB-α in the cytosol. In the presence of calcitriol, IkB-α mRNA is more stable. Calcitriol also decreases IKK phosphorylating activity on IkB-α, reducing IkB-α protein degradation and reducing production of TNFα.^76–78^ In addition, calcitriol induces MAPK phosphatase-1 (MKP-1), which dephosphorylates p38 and reduces p38 MAPK activation in monocytes/macrophages (Fig. 2F) and the production of TNFα and IL-6,^79^ a critical cytokine for the differentiation of Th17 cells. This leads to the phosphorylation of STAT3 and the activation of the transcription of retinoic acid-related orphan receptor. Furthermore, calcitriol promotes M2 macrophage polarization by increasing the expression of T-cell immunoglobulinmucin3 (Tim-3), resulting in a decrease in TNFα and IL-6 and an increase in IL-10^80^ (Fig. 2F). So, VitD indirectly inhibits Th17 cells and Th1 through the inhibition of production of IL-6 and TNFα by macrophages.

Altogether, these studies indicate that calcitriol inhibits EAE in a preventive approach and ameliorates EAE in a therapeutic approach. Clear evidence show that calcitriol reduces effector Th1 and Th17 while promoting Tregs, and decreases the infiltration of auto-aggressive T cells into the CNS. The reduced EAE depends on the expression of VDR in CD4^+^ T cells. VitD has both a direct and indirect immunosuppressive effect on immune cells through the increase in tolerogenic immune responses (IDO^+^-DCs, Treg and Breg) and in the anti-inflammatory cytokine IL-10. However, most studies focused on calcitriol, the active form of VitD, and relatively few animal model studies used cholecalciferol,^29,33,35^ the main VitD metabolite administered to multiple sclerosis patients.

## Immunomodulation in multiple sclerosis patients

While *in vivo* studies in EAE provided key molecular insights into the mode of action of VitD, several studies have analysed the effects of VitD on human lymphocytes from healthy donors or multiple sclerosis patients. Most studies determined the ability of VitD to modulate cell phenotype, proliferation and cytokine secretion *in vitro*. Several reports focused on the *in vivo* effects of VitD by directly assessing the levels of cytokines in serum or plasma of multiple sclerosis patients treated with high-dose VitD, or by analysing the proportions of key immune cell populations involved in multiple sclerosis pathophysiology (e.g. Treg, Th1, Th17).

**Figure 3:**
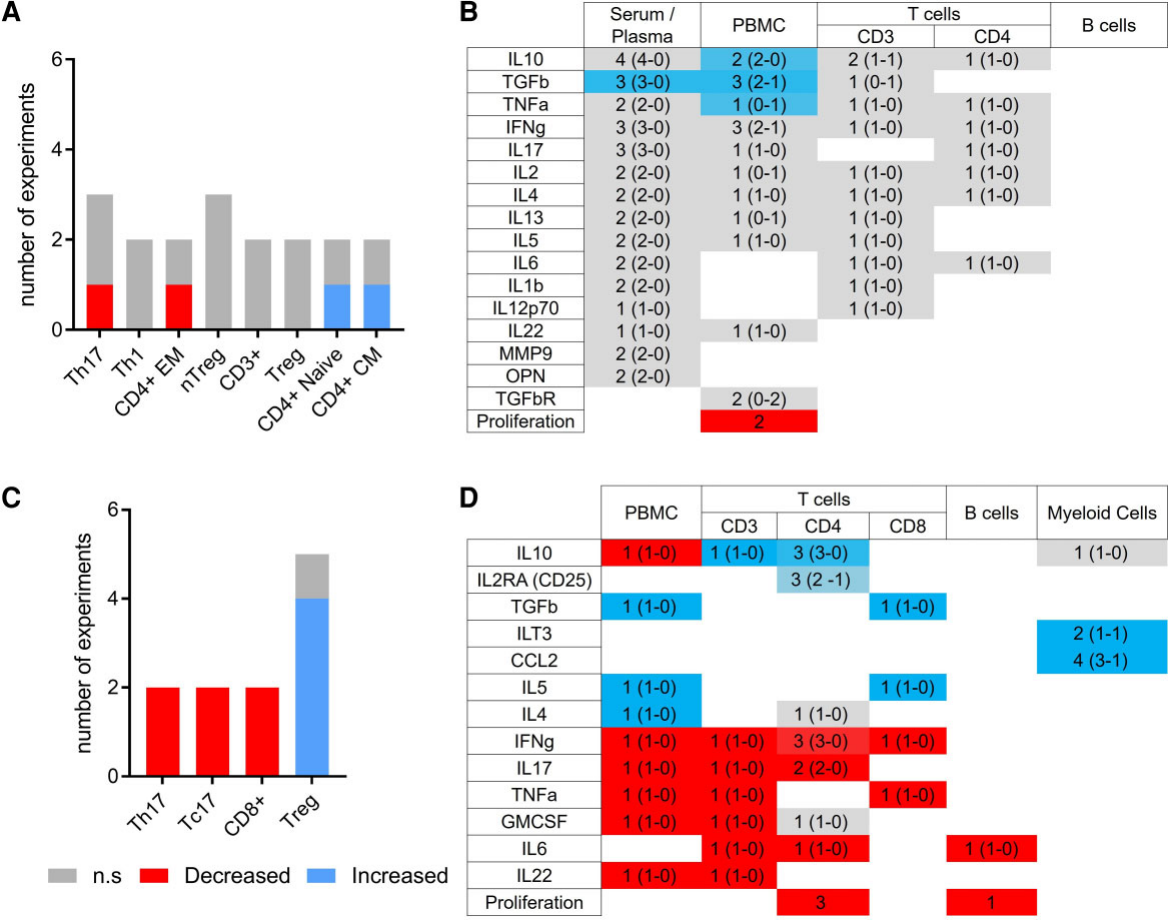
**Main effects and modulations of VitD treatment on immune cells in multiple sclerosis.** Effects and modulations of VitD investigated in more than one *in vivo* (**A** and **B**) and *in vitro* (**C** and **D**) studies. Graphs (**A** and **C**) represent the number of studies showing significantly increased (blue), decreased (red) or non-significantly modified (grey) cell populations after treatment by VitD. Heat-map (**B** and **D**) representing the modifications of circulating and secreted cytokines levels studied after VitD treatment/supplementation. Studies showing a significant increase (*n* = *a*), a significant decrease (*n* = *b*) or an absence of variation (*n* = *c*) were scored as follows: [(*a* × 2) − (*b* × 2)]/[(*a* × 2) + (*b* × 2) + c]. Red represents a global decrease, blue represents a global increase and grey non-significant or discrepant results. In each case, *X* (*Y*–*Z*) represents the total number of tests (*X*) and the number of tests realized using FACS/ELISA (*Y*) or PCR (*Z*).

An in-depth search of the recent literature (since 2010) identified 28 publications investigating the effects of VitD on peripheral immune cells and cytokines in multiple sclerosis patients. Fig. 3 summarizes the *in vivo* (Fig. 3A and B, Supplementary Fig. 1 and Table 1) and *in vitro* (Fig. 3C and D, Supplementary Fig. 1 and Table 2) studies analysing the changes observed in cell populations and expression of biomarkers in multiple sclerosis patients treated with high-dose VitD (range 7000–20 000 UI/day cholecalciferol) in therapeutic trials^81–94^ (Supplementary Table 1) or by adding active VitD (10–100 nM calcitriol) *in vitro* to peripheral blood mononuclear cells (PBMCs) or purified T cells obtained from multiple sclerosis patients^83,95–106^ (Supplementary Table 2).

In only one out of three *in vivo* studies looking at modifications of cell populations, VitD induced a significant decrease in circulating Th17 cells^82,84,85^ (Fig. 3A). Different studies focusing on other immune cell populations showed a significant decrease of CD4^+^ effector/memory T cells and a significant increase in circulating CD4^+^ naïve and CD4^+^ central memory T cells^85^ (Fig. 3A). Concerning the modifications of circulating and secreted cytokine levels in multiple sclerosis patients treated with high-dose VitD, a significant increase of IL-10, transforming growth factor beta (TGFβ) and TNFα was reported in total PBMCs^47,83,86,87^ and TGFβ in serum,^47,88,90^ as well as a significant decrease in proliferation in PBMCs^86,94^ (Fig. 3B). However, no significant effect of VitD was observed in cytokine secretion by T and B cells sorted by fluorescence activated cell sorting (FACS).

In contrast, *in vitro* studies showed a significant decrease in pro-inflammatory T-cell subsets, especially for those secreting IL-17 (Tc17 and Th17), and in CD8^+^ T cells^97,100^ (Fig. 1C, Supplementary Table 2). In four out of five studies, VitD induced a significant increase in circulating Treg.^95,97,98^ These studies indicate a significant increase of IL-2RA in CD4^+^ T cells, TGFβ and IL-5 in CD8^+^ T cells and in total PBMCs, ILT3 and CCL2 in myeloid cells, and IL-4 in total PBMCs^83,95–98,100,103–106^ and a significant decrease of pro-inflammatory cytokines such as IFN-γ, IL-17, TNFα, GM-CSF, IL-22, IL-6 in PBMCs, and in T-cell subtypes^95–98,100,101^ (Fig. 3D). VitD also induces a significant decrease of IL-6 in B cells.^102^ However, contrary to *in vivo* data, IL-10 is decreased in total PBMCs *in vitro*.^83^

Altogether, VitD decreases proliferation of CD4^+^ T cells and B cells and reduces cell populations secreting pro-inflammatory cytokines (IFN-γ, IL-17, TNFα, IL-6, GM-CSF, IL-22) while increasing the proportion of cell populations secreting anti-inflammatory cytokines (such as IL-10 and TGFβ). *In vitro*, the decrease in IL-17 secretion in CD4^+^ T cells is in accordance with the decrease in Th17 under VitD treatment (Fig. 3C and D). Similarly, the decrease of IFN-γ and TNFα secretion in CD8^+^ T cells reflects the decrease in CD8^+^ T cells, while the increase of IL-10 secretion and CD25 expression in CD4^+^ T cells correlates with the increase in Treg. However, *in vivo* studies provide inconclusive data on the modulation of immune cell populations as compared with the cytokine secretion regulation. However, CD8^+^ T cells, B cells and myeloid cells were not analysed in *in vivo* studies in multiple sclerosis patients and little is known on VitD effects on cytokine secretion profiles in specific subpopulations like Th1, Th2, Th17, Treg in this disease.

## VitD actions in the CNS

**Figure 4:**
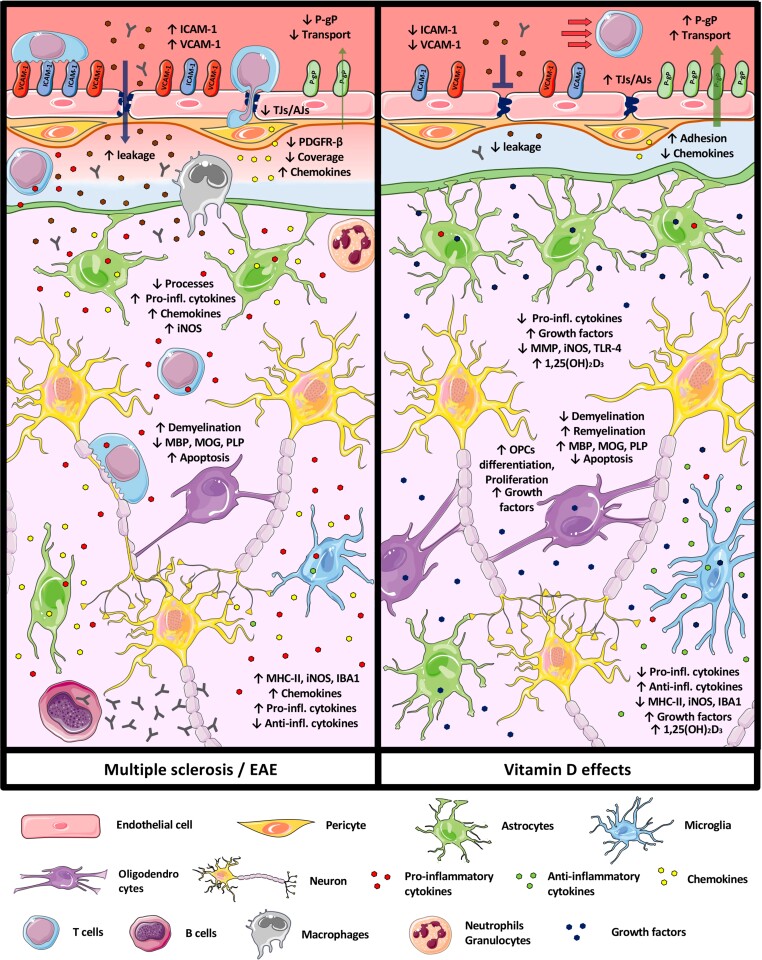
**VitD modulates different cellular and molecular mechanisms of CNS-resident cells and of the blood–brain barrier involved in multiple sclerosis pathology.** (Left) Schematic representation of the cellular and molecular mechanisms involved in multiple sclerosis/EAE pathology at the level of CNS-resident cells and the BBB. (Right) Schematic representation VitD impact on cellular and molecular mechanisms involved in multiple sclerosis/EAE pathology at the level of CNS-resident cells and the BBB. Changes in the cellular and molecular mechanisms are given as (↑) increased and (↓) decreased. Drawings of the individual cell types were adapted from Servier Medical Art (http://smart.servier.com/), licenced under a Creative Common Attribution 3.0 Generic License.

Immune regulations induced by VitD in the periphery may also protect the CNS from the inflammatory insult by local protection of the BBB and reduction of glial activation in the brain parenchyma. We particularly highlighted the effects of VitD on cellular mechanisms and cell types targeted by inflammation in multiple sclerosis (Fig. 4).

### At the BBB level

#### Endothelial cells

The CNS is an immune-privileged site with unique anatomic, cellular and molecular elements that limit both physiologic and pathophysiologic inflammation and preserve CNS homeostasis.^107^ The endothelial BBB of the CNS microvessels is composed of endothelial cells adjoined continuously by tight junctions (TJs), surrounded by pericytes and astrocyte end-feet that tightly ensheath the vessel, and possess the essential function of strictly controlling the cellular and molecular exchanges between the blood and the CNS.^70,108^ It is well established that BBB breakdown is an early hallmark of multiple sclerosis pathogenesis which is detected by gadolinium enhanced (Gd) MRI in the CNS of multiple sclerosis patients.^109,110^ Impaired BBB properties during multiple sclerosis are suggested by the observation of interrupted immunostaining for TJ and adherens junction (AJ) proteins, such as claudin-5 and VE-cadherin, respectively,^111–116^ as well as leakage of fibrinogen and immunoglobulin G (IgG) outside of blood vessels in post-mortem CNS tissue of multiple sclerosis patients^117^ (Fig. 4, Supplementary Table 3).

BBB endothelial cells express VDR, and VitD has been suggested to provide beneficial effects in multiple sclerosis by protecting BBB integrity as shown *in vitro* and *in vivo* (Table 1).

**Table 1:** Main cellular and molecular effects of VitD on CNS cells

	BBB (see Supplementary Table 3)	Pericytes (see Supplementary Table 4)	Astrocytes (see Supplementary Table 5)	Microglia (see Supplementary Table 6)	Oligodendrocytes (see Supplementary Table 7)
VitD metabolism					
VDR	↑ *in vitro*^118,119^ n.s. *in vitro*^120,121^ ↑ *in vivo*^122,123^	↑ *in vitro*^124,125^	↑ *in vitro*^126–128^ n.s. *in vitro*^121^	↑ *in vitro*^121,129–131^	↑ *in vitro*^132–134^
Cyp24a1	↑ *in vitro*^118^ n.s. *in vitro*^121^ ↑ *in vivo*^135,136^		↑ *in vitro*^121,127^	↑ *in vitro*^121,127,129^	
Cyp24b1			n.s. *in vitro*^121,127^		
Growth factors					
BDNF	↑ *in vivo*^137,138^				
NGF			↑ *in vitro*^139,140^		
Signalling					
p-AKT	↑ *in vitro*^141,142^				
p-ERK	↑ *in vitro*^141,142^				
NF-kb Activation	↓ *in vitro*^143,144^				
Cell viability	↑ *in vitro*^141,144^			n.s. *in vitro*^145–147^ ↑ *in vitro*^148^	
Leakage					
Claudin-5	↑ *in vitro*^143,144^				
ZO-1	↑ *in vitro*^143,144^ ↑ *in vivo*^149,150^				
TEER	↑ *in vitro*^143,144^				
Brain efflux	↑ *in vivo*^135,151^				
Permeability	↓ *in vivo*^26,122,137,138,149,150^				
Trafficking					
Mrp1	n.s. *in vivo*^120,152^				
P-gP	↑ *in vivo*^120,135,136,151,152^				
BCRP	n.s. *in vivo*^135,136^				
Oxidative stress					
NO-production	↑ *in vitro*^141,142^			↓ *in vitro*^145,147,148,153^ n.s. *in vitro*^154^	
ROS production	↓ *in vitro*^141,142^				
iNOS				↓ *in vitro*^129,131,148,155^ n.s. *in vitro*^153^	
SOD	↑ *in vivo*^137,156^				
Arg1				↑ *in vitro*^131,155^	
Cytokines					
IL-10				↑ *in vitro*^129,130^	
TNFa				↓ *in vitro*^131,145,147,153,154^ n.s. *in vitro*^148,155^ ↓ *in vivo*^157,158^	
IL-1b				↓ *in vitro*^131,148,155^	
IL-6				↓ *in vitro*^129,131,147,148,153–155^	
Myelin					
Myelination					↑ *in vivo*^34,159^
MBP					↑ *in vivo*^159,160^
Demyelination					↓ *in vivo*^34,159–162^
CNS insult					
Osteopontin	↑ *in vivo*^122,123^				
GFAP			↓ *in vivo*^163–166^ ↑ *in vivo*^161,167,168^ n.s. *in vivo*^169^		
Radio clinical parameters					
Brain oedema	↓ *in vivo*^122,137,150^				
Neurological deficit	↓ *in vivo*^122,137,150^ n.s. *in vivo*^170^				
New Gd-enhancing lesions	↓ multiple sclerosis Patients^171–173^ n.s. multiple sclerosis patients^86^				

Molecules/mechanisms/endpoints showing concordant variation after VitD stimulation in more than one study for each cell types are represented in this table. See Supplementary Tables 3–7 for experimental details. Arg1, Arginase-1; BBB, blood–brain barrier; BCRP, breast cancer resistance protein; BDNF, brain-derived neurotrophic factor; GFAP, glial fibrillary acidic protein; IL, interleukin; iNOS, inducible nitric oxide synthase; MBP, myelin basic protein; Mrp1, multidrug resistance-associated protein 1; NGF, neural growth factor; NO, nitric oxide; P-gP, permeability-glycoprotein; ROS, reactive oxygen species; SOD, superoxide dismutase; TEER, transepithelial/trans-endothelial electrical resistance; TNF-a, tumour necrosis factor alpha; VDR, vitamin D receptor.

For instance, 1,25(OH)_2_D_3_ prohibited the loss of TJs protein expression and decreased trans-endothelial electrical resistance (TEER) in brain microvascular endothelial cells (BMECs) incubated with TNF*α* or serum from RRMS patients *in vitro*.^143^ Furthermore, 1,25(OH)_2_D_3_ reduced endothelial cells apoptosis rate,^137,141,157^ suggesting a potential beneficial role of VitD in sustaining BBB endothelial cells survival during multiple sclerosis. With respect to *in vivo* data, 1,25(OH)_2_D_3_ treatment prohibited downregulation of zonula occludens-1 (ZO-) in spinal cord homogenates and reduced the extravasation of sodium fluorescein, Evans blue and IgG into the CNS of mice suffering from EAE.^26,149^ Similarly, different randomized clinical trials (RCTs) on the effect of daily oral high dose of cholecalciferol in multiple sclerosis patients showed significantly reduced numbers of new Gd or new/enlarging T2 lesions compared with placebo group.^171–173^

Additional evidence of VitD impact on preventing BBB breakdown comes from the research fields of ischaemic stroke and obesity. For instance, 1,25(OH)_2_D_3_ reduced the expression of matrix metalloproteinase-9 (MMP-9) and the permeability for fluorescein isothiocyanate (FITC)-dextran, as also increased TEER and TJs expression in a mouse brain endothelial cell line (bEnd3) cells under hypoxic conditions.^144^ Furthermore, 1,25(OH)_2_D_3_ treatment prior experimental stoke reduced Evans blue extravasation and infarct volumes in the CNS of different animal models for ischaemic stroke.^122,137,170^ Similarly, oral administration of cholecalciferol reduced BBB permeability for Evans blue in the hippocampus of obese rats,^138^ which do also possess an altered BBB.^174–177^ Furthermore, increased MMP-9 expression, IgG leakage and decreased immunostaining for TJs were observed in VitD-deficient rats after transient middle cerebral artery occlusion.^123^

Next to the presence of complex TJs, an additional property of the BBB is the presence of specific endothelial transporters, such as the efflux pump P-glycoprotein (P-gp), which actively removes unwanted compounds from the brain.^178^ Interestingly, P-gp expression was reduced in BMECs in active lesions of multiple sclerosis patients^179^ and P-gp transport function was significantly reduced during EAE in rats,^179^ suggesting reduced P-gp function at the BBB in multiple sclerosis. To the best of our knowledge, there are no studies investigating the impact of VitD on BBB transporters in multiple sclerosis. However, in the context of Alzheimer’s disease, different studies have demonstrated that VitD can modulate the expression of BBB transporters. In fact, 1,25(OH)_2_D_3_ increased P-gp expression and transport activity in BMECs *in vitro* and *in vivo*.^118,135^ Furthermore, 1,25(OH)_2_D_3_ pre-treatment reduced amyloid-β accumulation in the parenchyma of two different Alzheimer’s disease mouse models.^136^ Although the role of BBB transporters in multiple sclerosis pathogenesis still needs further investigation, these observations provide some evidence on how VitD could potentially be beneficial for BBB function in multiple sclerosis.

Besides BBB breakdown and changes in expression of BBB transporters, post-mortem brain tissues of multiple sclerosis patients have also shown increased adhesion molecules expression such as intercellular adhesion molecule (ICAM)-1 and vascular cell-adhesion molecule (VCAM)-1, accompanied by an increased infiltration of leucocytes into the CNS parenchyma.^114,180^ To this end, 1,25(OH)_2_D_3_ treatment reduced ICAM-1 and VCAM-1 expression on BMECs incubated with TNFα or serum from acute phase RRMS patients *in vitro*,^143^ highlighting the potential of VitD to modulate immune cell migration to the CNS by acting directly on the BBB endothelium. In line with this hypothesis, many studies have shown that VitD pre-treatment reduces immune cell infiltration into the CNS during EAE.^26,50,149,160,163,181,182^ However, the exact mechanisms by which VitD might modulate the BBB to control immune cell migration into the CNS still have to be elucidated. Furthermore, the effect of VitD on the expression of other important BBB adhesion molecules, which contribute to the recruitment of immune cells into the CNS have not been investigated yet.^114,183–187^

Taken together, these observations suggest a beneficial effect of VitD supplementation on BBB integrity during different neuroinflammatory processes. Still, there is currently no evidence of a direct effect of VitD on BBB endothelial cells *in vivo* during EAE nor in multiple sclerosis patients. Further investigations are required to clarify if VitD can exert a direct or indirect effect on the BBB endothelium and how this may affect the course of multiple sclerosis.

#### Brain pericytes

Pericytes are mural cells associated with the walls of blood capillaries^188^ and play a crucial role in sustaining BBB integrity.^189–195^ Indeed, BBB permeability is inversely proportional to pericyte coverage,^196,197^ and their loss leads to increased perivascular immune cells infiltration.^198,199^

The specific role of brain pericytes in EAE or multiple sclerosis has not been addressed in depth. Nevertheless, many studies have demonstrated the contribution of pericytes in mediating different neuroinflammatory processes.^199–203^ Furthermore, atypical symptoms and sudden death were observed in pericyte-deficient mice during EAE, underscoring a protective role of pericytes in neuroinflammation.^204^ During development, brain capillaries secrete platelet derived growth factor (PDGF)-β to recruit PDGF-receptor-beta (PDGFR-β) expressing pericytes to their wall.^190,205^ Mice suffering from EAE were reported to have reduced numbers of PDGFR-β^+^ pericytes, and lower expression of PDGFR-β expression in pericytes.^206,207^ With respect to multiple sclerosis patients, CNS biopsies showed that active lesions harbour higher numbers of proliferative and quiescent perivascular cells (including pericytes) compared with inactive ones, but that these are lower in chronic lesions when compared with healthy tissue.^208^ This indicates that the inflammatory status of the CNS plays an important role in determining the number of pericytes associated with microvessels.

Recent studies showed that brain pericytes lack the expression of the oxidative enzyme CYP27B1 and can therefore not synthesize 1,25(OH)_2_D_3_,^197,209,210^ suggesting that pericytes rely rather on paracrine or circulating 1,25(OH)_2_D_3_. Accordingly, only a few studies have investigated the effects of VitD on brain pericytes (Fig. 4 and Table 1, Supplementary Table 4).

Brain pericytes express VDR and its expression is increased upon 1,25(OH)_2_D_3_ exposure^124,125,143,210^ (Table 1). In addition, 1,25(OH)_2_D_3_ induced an anti-inflammatory phenotype in brain pericytes by downregulating the expression of chemokines like CCL2 *in* vitro.^210^ In line with this, 1,25(OH)_2_D_3_ increased the activity of the antioxidant enzyme γ-glutamyl transferase in brain pericytes *in vitro*.^211^ Furthermore, primary brain pericytes isolated from 1,25(OH)_2_D_3_ treated wild type (WT) mice showed reduced proliferation and migration *in vitro* but had increased adhesion to extracellular matrix proteins compared with cells isolated from VDR KO mice.^125^ The latter might ultimately help strengthening BBB integrity.

The effect of VitD on pericyte coverage in multiple sclerosis has to the best of our knowledge not yet been investigated. Nevertheless, it has been shown that vessels of tumours transplanted into VDR KO mice have less pericytes coverage compared with those in WT mice, suggesting that pericyte attachment might be partially dependent on VDR.^212^ In contrast, the endothelial cell/pericyte ratio was reduced in full retinal mounts of VDR KO mice compared with those of WT mice.^213^

Although the above-listed studies show variable effects of VitD on pericytes, further investigations are necessarily required to establish the therapeutic potential of VitD at this level.

### Biological effects on glial cells

#### Astrocytes

Astrocytes are among the most crucial modulators of brain function. They maintain neurotransmitters homoeostasis, cerebral blood flow, and provide essential nutrients and growth factors to the entire CNS.^214^ During development astrocytes play a major role in the maturation of the BBB by inducing for example the expression of TJs in CNS capillaries.^215^ Furthermore, astrocytes contribute to making the CNS an immune-privileged site by forming a second barrier called the glia limitans, which ensheaths the entire CNS parenchyma and, along with the BBB, prevents unrestricted entrance of immune cells.^107,216^

It has been shown that astrocytes are early responders in nascent white matter lesions of multiple sclerosis patients^217,218^ and in the CNS of mice after induction of EAE.^219–221^ Indeed, they can react promptly to inflammatory stimuli such as TNFα and IL-1β by amplifying neuroinflammation in a NFkB-dependent manner,^222^ and by recruiting leucocytes to the CNS via the expression of inflammatory chemokines, cell-adhesion molecules and nitric oxide synthase (iNOS).^223,224^ In multiple sclerosis, astrocytes attain an hypertrophic morphology and a reduced number of cytoplasmic processes, leading to the retraction of the glia limitans and thereby increasing the chance of immune cell infiltration in the CNS.^217^ On the other hand, activated astrocytes also exert beneficial effects by recruiting microglia and promoting clearance of myelin debris in the CNS of mice suffering from EAE.^225^ This directs us toward the possibility of a dual role of astrocytes in multiple sclerosis, by not only sustaining but also preventing the development of neuroinflammation.^216^

Many studies have shown that astrocytes express CYP24A1, CYP27B1 and VDR.^121,126,127,132,226–228^ These observations suggest that astrocytes can not only synthetize 1,25(OH)_2_D_3_ but also respond to it in an autocrine or paracrine manner. To this end, different studies on effects of VitD on astrocytes and their contribution in multiple sclerosis/EAE and other neuroinflammatory diseases have been conducted (Fig. 4 and Table 1, Supplementary Table 5).

Regarding inflammation, a recent *in vitro* study showed that 25(OH)D_3_ leads to a significant decrease in the expression of pro-inflammatory cytokines such as TNFα and IL-1β, as also TLR4 in activated astrocytes.^226^ Similarly, reduced TLR4 expression in the CNS of mice suffering from traumatic brain injury after combined 1,25(OH)_2_D_3_ and progesterone treatment was observed.^164^ Furthermore, a VitD_2_ analogue could inhibit NF-kB signalling in astrocytes of mice suffering from EAE, leading also to reduced clinical score.^163^ Taken together, these results point toward a beneficial effect of VitD treatment in multiple sclerosis by potentially reducing the pro-inflammatory response of astrocytes in the CNS.

With respect to CNS astrocytes density and GFAP expression, VitD treatment gave discrepant results in different neuroinflammatory animal models.^161,163–169^ For example, the VitD_2_ analogue reduced the populations of GFAP^+^ astrocytes in the spinal cord of mice suffering from EAE,^163^ while 1,25(OH)_2_D_3_ increased the number of GFAP^+^ astrocytes in the CNS of mice with cuprizone induced toxic demyelination.^161^ 1,25(OH)_2_D_3_ was also shown to rescue cell death by decreasing ROS production, iNOS and p53 activity in astrocytes *in vitro*.^229,230^ Similarly, VitD treatment decreased immunostaining for iNOS^+^ astrocytes in the CNS of EAE rats, suggesting VitD as a potential antioxidant treatment in multiple sclerosis, since ROS production is well established in the CNS of multiple sclerosis patients.^231^

Concerning cell survival, growth factors such as NGF and VEGF have been shown to be upregulated upon 1,25(OH)_2_D_3_ treatment in activated astrocytes *in vitro*.^128,226(p3)^,^139^ However, no changes in the expression of their receptors were observed.^126^ Furthermore, an analogue of 1,25(OH)_2_D_3_ decreased the proliferation, migration and metalloprotease activity of astrocytes *in vitro*.^232^

Altogether, these observations suggest a beneficial effect of VitD supplementation in reducing astrocyte activation and increasing their growth factor production in different models of multiple sclerosis.

#### Microglial cells

Microglia are the tissue-resident macrophages of the CNS parenchyma^233,234^ and play a key role in the brain and spinal cord by promoting synapse formation/refinement, oligodendrogenesis and myelin synthesis.^235^ In the CNS, microglia remain selfsustained by proliferation and build the first line of defence in the CNS parenchyma.^236^ In fact, microglia are very efficient in surveying the parenchymal environment for pathogens, cytokines, chemokines, neurotransmitters, neuromodulators and damaged cells.^237^ When a potential danger is recognized, microglia get activated assuming a more amoeboid-like appearance, and start clearing the surrounding environment by phagocytosis, an inherent characteristic of these cells that permits the maintenance of CNS homeostasis.^238,239^ Furthermore, they also secrete different growth factors such as TGF-β to promote cell survival.^240^

Microglia are recognized as a key player in many different inflammatory and neurodegenerative disorders. In the context of multiple sclerosis, microglia are abundant immune cells present in active lesions.^241^ Within active lesions, microglia play a critical role in antigen presentation, recruitment of T cells, ROS production and release of pro-inflammatory cytokines, which further amplify neuroinflammation.^239,242^ On the other hand, microglia are equally crucial for clearing myelin debris and enabling remyelination.^243^ These observations suggest that microglia play both a detrimental but also a beneficial role in multiple sclerosis pathology.

With respect to VitD, it has been demonstrated that microglia can synthetize calcitriol *in vitro*,^244^ since they express both CYP27A1 and CYP27B1.^121,127,129,145,245^ Along with BBB endothelial cells, pericytes and astrocytes, microglia also express VDR and can respond in either an autocrine or paracrine manner to calcitriol.^121,127,129,130,145,153,245^ Interestingly, a recent study showed that local synthesis of calcitriol by myeloid cells (microglia included) was essential for VitD-mediated EAE resistance.^246^ Several *in vitro* and *in vivo* studies in the broader context of neuroinflammation have also investigated the impact of VitD treatment on microglia (Fig. 4 and Table 1, Supplementary Table 6). Calcidiol and calcitriol can reduce microglia activation and antigen presentation by downregulating the expression of Iba1, MHC-II, CD86 and TLR-4 *in vitro* and *in vivo* in different neuroinflammatory models, including EAE.^131,146,149,155,158,162,165,246,247^ This was confirmed also by the observation of increased microglia activation in VitD-deficient mice.^146^ On the other hand, different *in vitro* and *in vivo* studies showed increased CD163, CD206, CD204 expression on microglia after calcitriol treatment, suggesting a switch toward an anti-inflammatory phenotype.^131,165,170^ Furthermore, calcidiol and calcitriol treatment reduced the production of pro-inflammatory cytokine such as IL-6, IL-1β, TNF-α, IFN-γ and MIP-1α, and on the other hand increased the expression of anti-inflammatory cytokines such as TGF-β1, IL-10, IL-4, IFN-α and IFN-β in microglia *in vitro* and *in vivo* in different neuroinflammatory models, including EAE.^129–131,147,148,153–155,158,165,248^

Along with a reduction in pro-inflammatory cytokine secretion, different *in vitro* studies have demonstrated that calcitriol can also reduce the production of ROS in microglia.^131,145,147,148,153^ This might result from the downregulation of iNOS expression in microglia after cholecalciferol, calcidiol and calcitriol treatment, as shown *in vitro* and *in vivo*.^129,131,148,155,165,230,247,249^ Furthermore, calcitriol increased microglia’s TGF-β expression^153,165^ pointing toward the possibility of a beneficial effect of VitD treatment in maintaining cell survival in the CNS of multiple sclerosis patients. Modulation of microglia phagocytosis by calcitriol has been also proposed by two different studies *in vitro*,^154,248^ however, with discrepant results.

Altogether, the current evidence suggests that VitD may alleviate the pro-inflammatory phenotype of microglial cells promoting an anti-inflammatory milieu in the CNS by directly modulating microglia activity during multiple sclerosis.

#### Oligodendrocytes

Oligodendrocytes are a type of neuroglial cells of the CNS, which provide support and insulation to axons by ensheathing them with multiple lamellae of their membrane, the myelin.^250^ They arise from oligodendrocyte precursor cells (OPCs)^251^ and are the last cell type generated in the CNS during development.^252^ Axon myelination is essential for fast and efficient signal transmission between neurons and proper neuronal function in the CNS.^253^ Furthermore, oligodendrocytes provide essential metabolic support to neighbouring cells by releasing different growth factors such as brain-derived neurotrophic factor (BDNF) and insulin-like growth factor-1 (IGF-1).^254^

Two different types of oligodendrocyte dysfunction, namely an immune-mediated oligodendrocyte dysfunction and a primary oligodendrogliopathy^255^ with subsequent demyelination and neurodegeneration accompanied by oligodendrocyte apoptosis have been observed in multiple sclerosis.^256^ In the first case, lymphocytes^257,258^ and auto-antibodies targeting myelin proteins (e.g. MBP, MOG and PLP)^259–261^ induce direct cytotoxicity to myelin and oligodendrocytes. In the second case, oligodendrocyte apoptosis as a response to stress precedes both inflammation and the recruitment of immune cells to the lesion site.^262^ Nevertheless, demyelinating plaques can sometimes undergo remyelination resulting in partial recovery of the neurological function in RRMS.^263^ This process is mainly sustained by OPCs which after activation, proliferate and migrate to the damaged area, and differentiate into mature oligodendrocytes to replace harmed myelin sheaths.^264^ On the other hand, in case of progressive multiple sclerosis, remyelination is impaired and neurological function is irreversibly lost.^265^

In contrast to microglia, oligodendrocytes lack the expression of CYP27B1 and are therefore unable to synthetize 1,25(OH)_2_D_3_.^121^ Nevertheless, oligodendrocytes and OPCs express VDR suggesting they can respond to calcitriol.^121,127,132–134,266^ To this end, different *in vitro* and *in vivo* studies investigated the impact of VitD treatment of oligodendrocytes and myelin in the context of multiple sclerosis (Fig. 4 and Table 1, Supplementary Table 7).

In the last two decades, multiple studies have demonstrated that cholecalciferol and 1,25(OH)_2_D_3_ treatment have a beneficial effect on myelination by reducing demyelination and increasing remyelination, as also restoring neurological function in the CNS in different animal models of multiple sclerosis including EAE.^34,159–162,266^ Indeed, animals suffering from neurodegeneration showed increased expression of MBP, MOG and PLP upon cholecalciferol or calcitriol treatment compared with vehicle control.^159,160,266,267^ Similarly, calcitriol treatment reduced OPC proliferation and lead to higher oligodendrocyte differentiation with consequent increase in MBP production *in vitro*.^133,266^ In addition, rats suffering from ethidium–bromide induced demyelination showed decreased caspase-3 activation in the CNS when treated with calcitriol, suggesting also a beneficial effect of VitD on oligodendrocyte apoptosis in multiple sclerosis.^159^ Along the beneficial effects of VitD on myelination, different *in vitro* studies showed that calcitriol could also increase the expression of growth factors (e.g. nerve growth factor in oligodendrocytes^132,134^ and BDNF in neural stem cells^133^ and therefore potentially sustain cell survival in the CNS).

Altogether, despite potential indirect effects of VitD on oligodendrocytes and the lack of evidence for a role of VitD on oligodendrocytes in multiple sclerosis brains, these results highlight VitD as a promising candidate to reducing demyelination (Table 1).

Globally, VitD protects the CNS from inflammation at the cellular level (BBB and glia) through the modulation of multiple mechanisms including cytokine and growth factors secretion, cell signalling, response to oxidative stress, BBB leakage and trafficking. The most robust effects of VitD in the CNS are summarized in Table 1.

## VitD therapy in multiple sclerosis patients

Despite all previous data about the potential immunological regulation and neuroprotective effects of VitD in multiple sclerosis models and multiple sclerosis patients, controversial results have been obtained from clinical trials evaluating VitD supplementation in multiple sclerosis patients. Kimball *et al*. evaluated tolerance and safety of escalating doses of active VitD ranging from 28 000 to 280 000 IU/week in an open-label study including 12 RRMS patients over a total of 28 weeks.^172^ Despite final mean serum concentrations of 25(OH)D of 386 ± 157 nmol/l, they did not observe any severe adverse events (SAEs) on this very small cohort. Since then, several SAEs (e.g. severe gastric symptoms, pseudo-brain tumour and seizures, tonic–clonic seizures, severe hypercalcemia) were observed at extremely high doses (100 000 IU/day for 1–36 months), suggesting that excessive supplementation with VitD can be dangerous despite a plateau concentration close to 400 nmol/l and that treatment duration might play a role in VitD toxicity.^268^ Moreover, little is known about the immunological effects of very high levels of circulating VitD. In animal models, the higher VitD dose, the higher immunomodulatory effect is generally observed, although excessive VitD treatment achieving serum levels over 250 nmol/L resulted in pro-inflammatory effects through lymphocyte activation and demyelination and myeloid infiltration into the brain, in addition to perturbations of phosphocalcic homeostasis.^269^

Since the original study by Mahon *et al*. in 2003, we identified more than 20 published case–control studies and double-blind RCTs aiming at evaluating the potential clinical and radiological benefits of VitD supplementation in multiple sclerosis patients.^47,81–94,171,173,270–276^ Some of these were recently reviewed and showed limited improvement in multiple sclerosis clinical and/or MRI features including fatigue, expanded disability status scale, relapses, T2-lesions, non-evidence of disease activity (NEDA-3) and in retinal nerve fibre layer thickness.^21,277^ These trials also evaluated safety of VitD supplementation at more physiological dosages (<15 000 IU/day) and did not observe a significant increase of AEs or SAEs in VitD-treated groups compared with placebo groups (CHOLINE,^275^ SOLAR^171^). Importantly, phosphocalcic homeostasis seemed respected at this level of supplementation, as no cases of calciuria and hypercalcemia were observed (SOLAR).

The safety range of VitD levels has been defined by an expert consensus in 2010 as of 30–100 ng/ml (equivalent to 75–250 nmol/l), considering that in healthy subjects who have spent prolonged periods in a sunny environment, measured 25(OH)D concentrations rarely exceed 100 ng/ml.^278^ In populations with light skin frequently exposed to the sunlight, the upper limit of circulating VitD levels is located between 75 and 150 nmol/l, providing an optimal range to reach after VitD supplementation, as defined by experts of bone and calcium homeostasis in order to prevent osteoporosis and fractures.^279^ However, so far, there are no recommendations for multiple sclerosis patients on potential VitD supplementation to achieve optimal levels for immune modulation and clinical effect.

**Figure 5:**
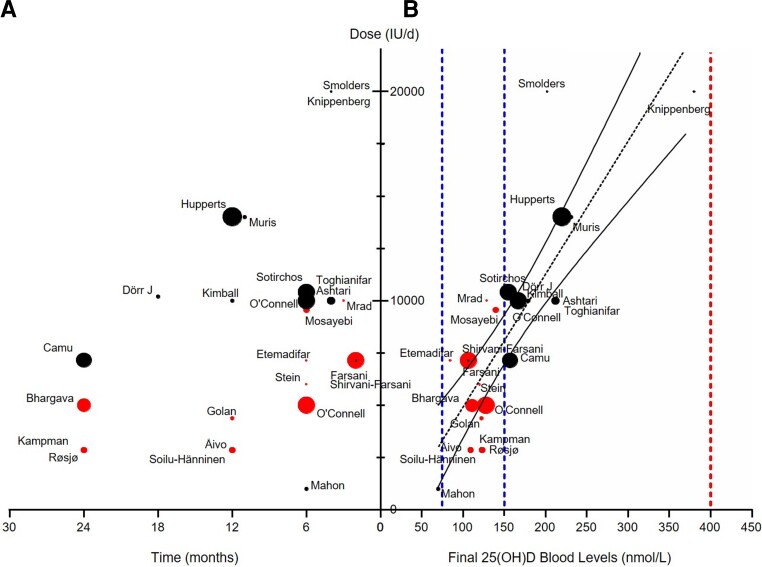
**Correlation between daily dose of VitD and the final blood levels of 25(OH)D from different Phase 2 and Phase 3 RCTs in RRMS patients.** (**A**) Representations of the different clinical studies on the effect of VitD in multiple sclerosis patients according to the dose administered (IU/day) as a function of time (months). (**B**) Representations of the different clinical studies in multiple sclerosis patients according to the final 25(OH)D blood levels (nmol/l) as a function of time (months). Dots are proportional to the number of patients in each study. Red dots represent studies with final blood VitD levels in the range 75–150 nmol/l as determined by Souberbielle *et al*., 2019; other studies are represented in black. Linear regression shows a significant correlation between daily dose of VitD and the final blood levels of 25(OH)D [*r*^2^ = 0687; 25(OH)D = daily dose/63.5 + 22.5].

In order to define VitD dosing to achieve optimal VitD therapeutic range after supplementation, we analysed 24 therapeutic trials accounting for 742 treated patients and providing their final VitD levels. When plotting the time of exposure with the daily dose of VitD supplementation of the treated group, we observed that protocols were very heterogeneous with treatment duration ranging from 2 to 24 months and dosages ranging from 1000 to 20 000 UI/day (Fig. 5A). However, we observed a strong correlation between the daily dose and the final circulating VitD levels of treated patients (Spearman *r* = 0.803, *P* < 0.0001, Fig. 5B), indicating that a daily dose of VitD between 3000 and 11 000 IU for 3–24 months achieves final blood VitD levels within the 75–150 nmol/l recommended range (red dots, Fig. 5A and B). Even at the higher doses used in the SOLAR study^171^ where patients received 14 000 UI/day for 12 months, VitD levels above 200 nmol/l were obtained without occurrence of SAEs after 1 year. This potentially increases the safety of higher VitD supplementation reaching up to 250 nmol/l for short periods. However, the exact concentrations of VitD in multiple sclerosis needed to properly restore immune homeostasis over the long term remains to be determined as well as the optimal risk/benefit balance for VitD supplementation. Given the weak clinical and radiological effects of VitD treatment in short-term RCTs involving multiple sclerosis patients, further analysis of the *in vivo* immunological efficacy of VitD are warranted to refine the objectives of chronic VitD supplementation in multiple sclerosis patients.

## Discussion and perspectives

While it is indisputable that VitD exerts strong immunomodulatory effects, it is still debated whether VitD supplementation is efficacious in multiple sclerosis. Data from animal models and *in vitro* studies provide important information on VitD effects to optimize its clinical application. Despite the large number of studies published in the field of multiple sclerosis so far, sound review of the literature revealed that analysis of VitD modulation of the immune system only focused on subpopulations of PBMCs and cytokine secretion, principally by FACS, ELISA and RT-PCR. FACS relies on designed panels of antibodies coupled to serial wavelength fluorophores to target markers of cell surface (and also intracellular markers) and analyse ± sort cells of interest, providing an efficient tool to study immune cell populations. However, when dealing with complex immunological diseases like multiple sclerosis, limited numbers of lasers and channels does not provide the ability to study subpopulations of a given sample in a row. The recent development of high-parameter FACS equipped with 4–7 lasers allows the detection of up to 18–30 markers in each cell that is much more in line with the investigation of several subpopulations.

New and innovative techniques developed recently could also be of high interest to further discriminate lymphocyte subsets of importance in multiple sclerosis pathophysiology, regulation of which by treatments is mandatory to efficiently reduce inflammation and disease activity. Taking advantage of antibody coupling to non-radioactive isotopes of heavy metals that can be detected by mass spectrometry, cytometry by time of flight (CyTOF) enhances the study and analysis of very specific types of cells.^280^ This technique allows the detection of a large number of cell types and the assessment of the immune cell activation state (around 60 markers) and overcomes the limitations of the use of fluorophores in flow cytometry. Using this technique, Couloume *et al*.^281^ detected an increased abundance of a T-bet expressing B-cells subset and an increased abundance of a CD206^+^ classical monocytes subset in the blood of multiple sclerosis patients with early multiple sclerosis. Johansson *et al*.^282^ used CyTOF and identified an multiple sclerosis-associated B-cell population using an unsupervised clustering of all CSF cells characterized by the expression of CD49d, CD69, CD27, CXCR3 and also found that proteins involved in neural plasticity were reduced in multiple sclerosis. Comparing multiple sclerosis to other inflammatory and non-inflammatory conditions of the CNS, Galli *et al*.^283^ identified an expanded T-helper cell subset characterized by the expression of GM-CSF and the C–X–C chemokine receptor Type 4 (CRCX4), revealing Th cells as the major contributor to GM-CSF production in the peripheral immune compartment of multiple sclerosis patients, with highest GM-CSF production in the effector memory fraction. Using intracellular staining after permeabilisation of cells, they combined surface and intracellular markers for mass cytometry and showed that the vast majority of GM-CSF producing Th cells co-expressed TNFα and IL-2.^283^ Although intracellular markers can be used for additional analyses, one limitation of CyTOF is that cells are directly discarded at the end of the analysis and cannot be sorted for complementary functional tests *in vitro*. Another limitation of CyTOF is the important signal variation over time and among centres, precluding multicentre studies unless a reference sample is used to normalize the data and ensure reliable comparisons. Finally, CyTOF is limited by the targeted investigation of specific cell populations and markers using a panel of antibodies designed by the experimenter, precluding discovery of novel markers of new cell subtypes potentially playing an important role in multiple sclerosis. Despite these limitations, CyTOF emerges as a very innovative technique with a high potential to investigate immune regulation induced by VitD in multiple sclerosis patients.

Another approach to decipher changes in immune cell profiles and activation is RNA sequencing (RNAseq). This technique, relying on the identification and quantification of gene transcripts (coding RNA) in each sample is used to analyse qualitatively and quantitatively gene expression changes. It can also be used to investigate non-coding RNAs potentially playing the role of regulators of gene expression. Comparing the linear and circular transcriptomes of leucocytes from 50 multiple sclerosis patients and 20 healthy controls (HC), Iparraguirre *et al*.^284^ found 689 upregulated and 683 downregulated transcripts in multiple sclerosis patients, most of them related to immune processes including neutrophil activation involved in immune response, neutrophil degranulation and granulocyte activation. Investigating circular RNA (circRNA) known as non-coding RNA regulating various immune responses, they observed a global upregulation of circRNAs in multiple sclerosis patients compared with HCs, with similar circRNA profiles between multiple sclerosis types. Long non-coding RNAs (lncRNAs) were also investigated in multiple sclerosis patients versus HCs by Han *et al*.,^285^ who identified 1438 downregulated and 945 upregulated lncRNAs, some of which were found to influence the antigen processing/presentation and MAPK signalling pathway in multiple sclerosis. To provide more informative data regarding immune regulation, RNAseq can be used to investigate the modifications of specific lymphocyte populations sorted by FACS. Fernandes *et al*.^286^ reported a detailed gene expression by RNAseq profiling analysis of FACS-sorted CD4^+^ and CD8^+^ T cells from HCs, RRMS and SPMS patients. Their analysis revealed 149 differentially expressed genes in both cell types, confirming previous findings (human-leucocyte antigen G) and discovering several novel genes and processes involved in multiple sclerosis, like IgG heavy chain and neurofilament-light chain. Looking at the effects of VitD in THP-1 cells (the human acute monocytic leukaemia cell line) by RNAseq, Neme *et al*.^287^ identified 1284 out of 14 402 expressed genes (8.9% of the expressed transcriptome of THP-1 cells) regulated by 1,25(OH)2D_3_. In human PBMCs from HCs exposed to a bolus of VitD (80 000 UI), they also observed that 702 genes were significantly regulated, including genes involved in general protein translation, monocyte differentiation and cellular growth control, suggesting that this approach could be applied to multiple sclerosis patients.^288^

More recently, single cell RNAseq (scRNAseq) has emerged as a powerful method to analyse modifications of cell populations and activation state. Compared with CyTOF, scRNAseq data are much more highly dimensional. Compared with bulk RNAseq that provides data from a collection of mixed cell populations to which we cannot point to the individual contribution, ScRNAseq directly reveals all the transcripts expressed by a cell, thus ensuring a qualitative and quantitative analysis of gene changes of all cell subsets of the sample. In EAE, using a combination of scRNAseq and other techniques, Wheeler *et al*.^289^ identified a reduced NRF2-driven gene expression and increased MAFG and MAT2α signalling in astrocytes, potentially promoting pro-inflammatory genomic programmes in astrocytes via GM-CSF, produced by inflammatory T cells recruited to the CNS in multiple sclerosis and EAE. Using scRNAseq to establish a detailed molecular description of microglia from HCs and multiple sclerosis patients, Masuda *et al*.^290^ observed that the microglial profile from HCs was enriched for transcripts indicative of a homeostatic phenotype, while multiple sclerosis patients showed multiple activated subsets potentially indicating engagement with infiltrating autoreactive T cells, matrix remodelling or reduction of cytotoxic CD8^+^ T-cell functions. Esaulova *et al*.^291^ used scRNAseq to profile individual cells of CSF and blood from two subjects with RRMS and one with anti-MOG associated disorder and identified myeloid cell types expressing a microglial signature. Finally, using scRNAseq and FACS coupled to bulk RNAseq, Ramesh *et al*.^292^ analysed paired CSF and blood B-cell subsets from multiple sclerosis patients, showing that B cells primarily clustered based on their subset type and the body compartment from which they were isolated. Independent analyses across technologies demonstrated that NF-κB and cholesterol biosynthesis pathways were activated, and specific cytokine and chemokine receptors were upregulated in CSF memory B cells.

Altogether, scRNAseq has a high potential to investigate the effects of VitD in immune cells in multiple sclerosis patients. However, scRNAseq has limitations, especially concerning the identification of cell subtypes according to the gene transcripts alone, especially concerning CD4^+^ T cells that show very little expression of clusters of differentiation genes. To overcome this caveat, the use of antibodies generally used for characterization of cell subtypes by FACS has been barcoded (adjunction of a specific RNA sequence on the Fc fragment of the protein) to ensure faithful selection of the target population for genomic analysis. This method called Cellular Indexing of Transcriptomes and Epitopes by sequencing (CITE-seq) allows accurate characterization of both phenotype and transcriptome in a scRNAseq experiment.^293–295^

All these data strongly support the accuracy of CYTOF/CITE-seq and scRNAseq to analyse the effects of VitD on the whole PBMC population to improve our understanding of the pleiotropic effects of VitD while keeping detailed analysis of key populations modified by VitD.

## Conclusion

Despite conflicting results, the overall effect of VitD supplementation in multiple sclerosis patients and animal models of multiple sclerosis results in a global modulation of inflammation. Inconclusive results from the clinical studies analysing the effect of VitD supplementation as add-on therapy in small numbers of multiple sclerosis patients or during short periods do not exclude a potential protective effect *in vivo*. Despite an excellent clinical safety profile of VitD, excessive doses are associated with SAEs, limiting the therapeutic range of VitD much below the high doses generally used *in vitro*. Finally, well-powered multicentre randomized controlled trials of VitD monotherapy versus placebo are still needed to bring significant data highlighting the therapeutic effectiveness of VitD in multiple sclerosis, and to refine the optimal posology to reach clinical effects and allow generalization of VitD supplementation in multiple sclerosis. The ongoing RCT D-lay multiple sclerosis (NCT01817166) included 316 multiple sclerosis patients within 3 months from disease onset to show the ability of high-dose cholecalciferol versus placebo for 2 years to reduce disease activity. This study will also provide useful biological samples to decipher the complex modifications of immune cell hierarchy and functions by coupling high-throughput technologies such as high-parameter flow cytometry and CITE-seq.

Data sharing is not applicable to this article as no new data were created or analysed in this study. Statistical analysis is not applicable to this article.

## Supplementary Material

fcac171_Supplementary_Data

## Abbreviations

BBB =: blood–brain barrier
CITE-seq =: Cellular Indexing of Transcriptomes and Epitopes by sequencing
CyTOF =: cytometry by time of flight
DCs =: dendritic cells
EAE =: experimental autoimmune encephalomyelitis
FACS =: fluorescence activated cell sorting
ICAM-1 =: intercellular adhesion molecule-1
IFN-γ =: interferon gamma
IgG =: immunoglobulin G
IL =: interleukin
KO =: knockout
MBP =: myelin basic protein
MHC =: major histocompatibility complex
MMP9 =: matrix metalloproteinase-9
MOG =: myelin oligodendrocyte glycoprotein
NFAT =: nuclear factor of activated T cells
NF-kB =: nuclear factor-κB
OPCs =: oligodendrocyte precursor cells
PBMC =: peripheral blood mononuclear cells
PDGF =: platelet derived growth factor
P-gp =: P-glycoprotein
PLP =: proteolipid protein
RCTs =: randomized clinical trials
RNAseq =: RNA sequencing
RRMS =: relapsing-remitting multiple sclerosis
RXR =: retinoid-X-receptor
SAEs =: severe adverse events
scRNAseq =: single cell RNA sequencing
SPMS =: secondary progressive multiple sclerosis
TEER =: trans-endothelial electrical resistance
TJs =: tight junctions
TGF-β =: transforming growth factor beta
TNFα =: tumour nuclear factor alpha
VCAM-1 =: vascular cell-adhesion molecule-1
VDR =: vitamin D receptor
VDRE =: vitamin D receptor element
VitD =: vitamin D
WT =: wild type
