# Supplementary material for: Full spectrum of vitamin D immunomodulation in multiple sclerosis: mechanisms and therapeutic implications
Source: Brain Commun. 2022 Jun 30;4(4):fcac171. doi: 10.1093/braincomms/fcac171 (PMC9260308; doi:10.1093/braincomms/fcac171)
Supplement: fcac171_Supplementary_Data [file fcac171_supplementary_data.docx]

***
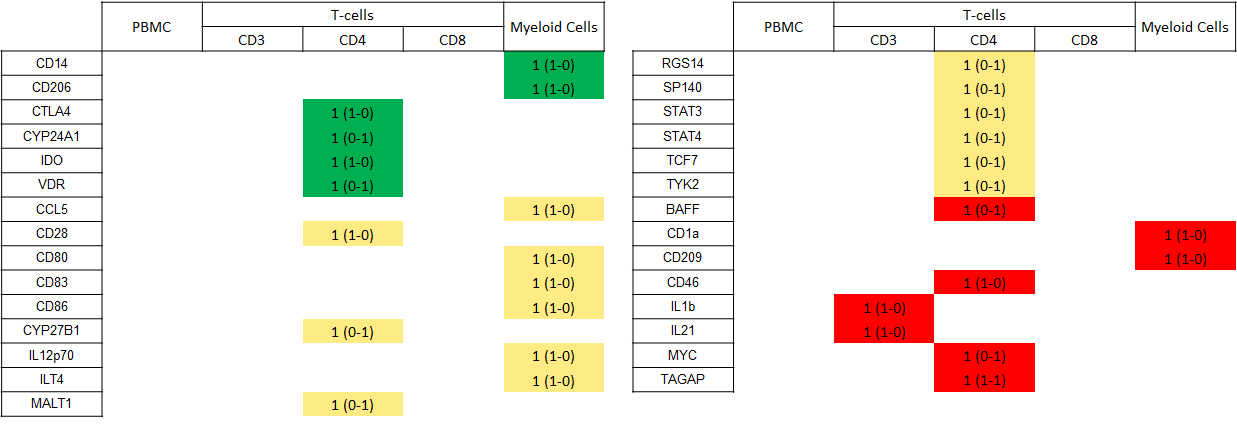
***
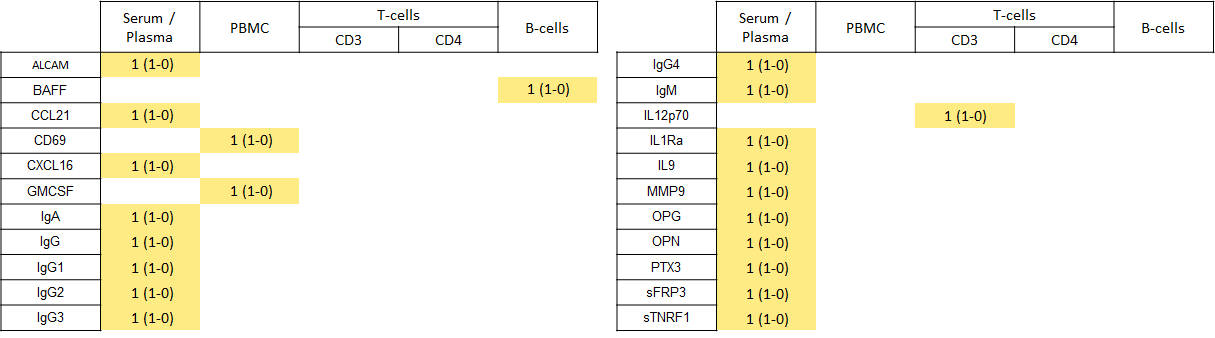


D

C

B

A

**Supplementary Figure 1**. Heat-Map representing the modifications of expression of biomarkers studied in *in vitro* (A,B) and *in vivo* (C,D) studies. Studies showing a significant increase (n=a), a significant decrease (n=b) or an absence of variation (n=c) were scored as follows: ((a*2)-(b*2))/((a*2)+(b*2)+c). Red represents a global decrease, green represents a global increase and yellow non-significant or discrepant results. In each case, X (Y-Z) represents the total number of tests (X) and the number of tests realized using FACS/ELISA (Y) or PCR (Z).

|  | Population characteristics | | | Vit D (cholecalciferol) supplementation | | | | | | Immune effects of Vitamin D | | | |
| --- | --- | --- | --- | --- | --- | --- | --- | --- | --- | --- | --- | --- | --- |
|  | MS (F/M) | DMD | Relapse free (months) | Dose  UI/  week | Duration  (months) | Control | Vit D isoform | Method | Mean Vitamin D levels  (baseline – end) | Cell population | Methods | Cytokines/Chemokines | Immune cells |
| Åivo et al., 2015 | 30 (18/12) | IFN-beta-1b | 1 | 20,000 | 12 | Placebo | 25(OH)D2 + 25(OH)D3 | RIA | 54 - 109 | Serum | ELISA | ↑ LAP (TGF-β),  IFN-γ, IL-17A, IL-2, IL-10, IL-9, IL-22, IL-6, IL-13, IL-4, IL-5, IL-1β, TNF-α |  |
| Ashtari et al., 2015 | 44 (35/9) | IFN-beta | 1 | 70,000 | 3 | Placebo | 25(OH)D3 | ECL | 28 - 85 | Serum | ELISA | IL 10 |  |
| Farsani et al., 2015 | 32 (27/5) | No | MD | 50,000 | 2 | Before/ after | 25(OH)D2 + 25(OH)D3 | ELISA | 20 - 60 | CD3 | qRT- PCR | **↑** IL 10 mRNA *, TGF B1 mRNA, |  |
| Golan et al., 2013 | 20 | IFN-beta | MD | 30,590 | 3 | 800 IU/day | 25(OH)D2 + 25(OH)D3 | CLIA | 48 - 122 | Serum | ELISA | IL 17, IFN-γ, IL 10 |  |
| Kimball et al., 2011 | 25 | IFN-beta, GA, none | 2 | 70,000 | 12 | maximum 4000 IU/D  open label | 1,25(OH)2D3 / 25(OH)D2 + 25(OH)D3 | RIA | 155 – 184 /  78 - 179 | CD3 | ELISA, [3H] thymidine incorporation | IL-1 béta, IL-4, IL-5, IL-6, IL-10, IL-12p70, and IL-13, IFN-γ, and TNF-α, MMP-9, tissue inhibitor of MMP-1, kallikrein-6, CRP, OPN | ↓ proliferative response of lymphocyte for neuron antigens (Ex-2 and MBP) |
| Knippenberg et al., 2011 | 15 | IFN-beta 1a or 1b | 1,5 | 140,000 | 3 | Before/ after | 25(OH)D2 + 25(OH)D3 | RIA | 50 - 380 | Plasma, B cells | Nephelometry, ELISA,  FACS | IgG1, IgG2, IgG3 or IgG4, IgM and IgA, BAFF levels, | Total B cells (CD19+), proportions of circulating naive B cells (CD19+CD276+), memory B cells (CD19+CD27+), unswitched memory B cells (CD19+CD27+IgD+), switched memory B cells (CD19+CD27+IgD-) |
| Mahon et al., 2003 | 17 | MD | MD | 7,000 | 6 | Placebo | 25(OH)D2 + 25(OH)D3 | RIA | 17 - 28 | PBMCs and serum | ELISA,  Semi-quantitative PCR | ↑ TGF-β1  IL-2 mRNA, IL13 mRNA, TNF-α mRNA, IFN-γ mRNA |  |
| Mosayebi et al., 2011 | 26 (17/9) | IFN-beta-1a | During study | 70,000 | 6 | Placebo IM | 25(OH)D2 + 25(OH)D3 | Enzyme immunoassay | 25 - 140 | PBMCs | ELISA, MTT assay method | ↑ TGF-β1, IL 10,  IFN γ | ↓ lymphoproliferation of PBMC |
| Mrad et al., 2017 | 21 | IFN-beta-1a or 1b | 1 | 10,000 | 3 | Before after | 25(OH)D2 + 25(OH)D3 | Roche Diagnostics total assay | 16 - 51 | CD4+ | ELISA, FACS | IFNγ**,** IL-17, TNFα, IL-10, IL-6, IL-4, IL-2 | Naïve T-CD4 (CD4+RO−CCR7+), central memory T-CD4(CD45CD4+CR7+), effector memory T-CD4 (CD45RO+-CD4+7−), Th17 (CCR6+- IL23R+CD161+IL-17+GMCSF+), Th1, Treg, CD3+ CD4- |
| Muris et al., 2016 | 23 | IFN-beta-1a | 1 | 49,000 for 4 weeks, followed by 98,000 | 11 | Placebo | 25(OH)D2 + 25(OH)D3 | RIA | 60 - 231 | PBMCs and serum | ELISA, FACS | ↑ TNF α,  IL- 10, IL-4, IL-5, IFN γ, IL-17, IL-22, GMCSF, LAP, IFNγ/IL5 | Breg (CD24hiCD38hiCD19+IL10+), CD39+ nTreg (CD25+CD127-CD39+), IFNγ+ Th cells, IL17+ Th cells, IL22+ Th cells, IL4+ Th cells,  iTreg (CD4+IL10), nTregs : CD25+CD127- ; CD25+CD127-FoxP3+ ; CD25+FoxP3+  memory nTreg (CD4+CD25+CD127-CD45RA-), Th 1 ou 17 GMCSF+, TNFα+ Th cells, Th1/Th2 ratio |
| Røsjø et al., 2015 | 36 (25/11) | IFN-beta, GA, NTZ | MD | 20,000 | 24 | Placebo | 25(OH)D2 + 25(OH)D3 | MSpec | 56 - 123 | serum | ELISA | ALCAM, CCL21, CXCL16, IL-1 Ra, MMP9, OPN, OPG, PTX3, sFRP3, sTNF R1, TGF β 1 |  |
| Shirvani-Farsani et al., 2015 | 31 (26/5) | MD | 2 | 50,000 | 2 | Before after | 25(OH)D2 + 25(OH)D3 | Enzyme immunoassay | 12 – 43  (12 – 44 / 12 – 33) | PBMCS | qRT- PCR | ↑ TGF-β2 mRNA,  TGF-βRI mRNA,TGF-βRII mRNA |  |
| Smolders et al., 2010 | 15 (8/7) | IFN-beta-1a or 1b | 1,5 | 140,000 | 3 | Before after | 1,25(OH)2D3 / 25(OH)D2 + 25(OH)D3 | RIA | 146 – 202 / 50 - 380 | CD4+ | FACS | CD69 | ↑ Treg ou Tr1 ou Th2 : CD4+IL10+  ↓ ratio IFN-γ+CD4+ and IL-4 +CD4+/ (Th2 >Th1)  T cells (CD3) in circulation, T activated (CD25+FoxP3+), Th1 (CD4+IFN gamma+), Th17 (CD4+IL17+), Th2 (CD4+IL4+), Treg (CD25+CD127-CD4+), Treg memory (CD25+CD127-CD45RA-), Treg Naive (CD25+CD127-CD45RA+), Tresp cell proliferation, Treg suppressive function |
| Sotirchos et al., 2016 | 19 (14/5) | IFN-beta, GA, NTZ | 1 | 72,800 | 6 | 800 IU/D | 25(OH)D2 + 25(OH)D3 | CLIA | 27 - 60 | PBMCs | FACS |  | ↑ naive CD4+ (D45RA+ 4+D4+ls +), CM CD4+,  ↓ EM CD4+, Th17 (CD4+IL-17+), Th17 ou MAIT (CD161+CD4+), CD85j+ proportion of CD8+ cells,  IFN-g+CD4+ T cells, IFN-g+IL-17 + CD4+ T cells, |
| Toghianifar et al., 2015 | 44 (35/9) | IFN-beta | 1 | 70,000 | 3 | Placebo | 25(OH)D3 | ECL | 28 - 85 | serum | ELISA | IL 17 |  |

**Supplementary Table 1: Effects of VitD on MS patients immune cells *in vivo*.**

The effects of Vitamin D (cholecalciferol) are given as ↑ increased; ↓ decreased; not affected when not specified. * A significant increase of IL 10 mRNA occurred only when vitamin D plasmatic levels after treatment raised more than 20 g/ml.

ALCAM : Activated leukocyte cell adhesion molecule, BAFF : B cell activating factor, CCL : Chemokine (C–C motif) ligand, CLIA : Chemiluminescent Immuno-assay, CXCL : Chemokine (C–X–C motif) ligand, DMD : disease modifying drug, ECL : Electrochemiluminescence, ELISA : enzyme-linked immunosorbent assay, FACS : fluorescence-activated cell sorting analysis with cytometry, GA : glatiramer acetate, GM-CSF : Granulocyte-macrophage colony-stimulating factor, IL: interleukin, INF :interferon, Ig : Immunoglobulin, LAP : latency activated protein of TGF β, MSpec : Mass Spectroscopy, MD : missing data, MMP :Matrix metallopeptidase, NTZ : natalizumab, OPG : Osteoprotegerin, OPN : osteopontin, PBMC : peripheral blood mononuclear cells, PTX3 : Pentraxin 3, qRT-PCR : quantitative real-time polymerase chain reaction, RIA : radio Immuno-assay, sFRP3 : Secreted frizzled-related protein 3, sTNF R1 : Soluble tumor necrosis factor receptor 1, TGF :transforming growth factor, TNF : tumor necrosis factors,

|  | Population  (female/male) | Culture conditions | | | | | | Immune effects of Vitamin D | | |
| --- | --- | --- | --- | --- | --- | --- | --- | --- | --- | --- |
|  |  | Cells type used | Vit D metabolite | Dose | Duration (days) | Control | Stimulation | Detection methods | Cytokines/Chemokines | Immune cells |
| Berge et al., 2016 | RRMS (8) | T CD4+ | 1,25(OH)2D3 (calcitriol) | 10 nM | 1 | vehicle control (ethanol) | αCD3/ CD28 coated beads | qRT-PCR, FACS | ↑ CD25, IL2RA mRNA, CYP24A1 mRNA (24 hydroxylase),  ↓ TAGAPprot, TAGAP mRNA |  |
| Bartosik et al., 2009 | RRMS (26) F18/M8 | Mo-DCs | 1,25(OH)2D3 (calcitriol) | 1nM | 2 | without | TNF α | ELISA, FACS | ↑ CD14, CD206, CCL2  ↓ CD1a, CD209, IL-23/IL-12p40, IL-23/IL-12p40,  IL10, IL12p70 and CCL5, CD83, CD80, CD86, |  |
| Correale et al., 2009 | RRMS (20) | T CD4+ | 1,25(OH)2D3 (calcitriol) | 10 nM | 3 | 24,25 (OH)2 Vitamin D  and 25,26 (OH)2 Vitamin D  or without | 1 mg/ml of plate-bound anti-CD3 mAb | [3H] thymidine incorporation, qRT-PCR  ELISA | ↑ VDR mRNA, IDO mRNA, IL-10  ↓ IL-6 and L-17,  CYP27B1 mRNA (1-hydroxylase), IFN γ, IL4 | ↑ fraction T reg (CD4+CD25+FoxP3+)  ↓ proliferation of CD4+ T cells |
| Correale et al., 2010 † | RRMS (32) 17F/15M | T CD4+ | 1,25(OH)2D3 (calcitriol) | 10 nM | 3 | without | 9 mg/ml PHA or myelin peptide-specific TCLs + immobilized anti-CD3 mAb | [3H] thymidine incorporation,  ELISA, FACS,  qRT-PCR | ↑ IL-10,  *↓ IFN γ,* IL-17, CYP24A1 transcript induction (24 hydroxylase mRNA), CYP27B1 mRNA (1-hydroxylase), VDR mRNA | ↑ Fraction T reg (CD4+CD25+FoxP3+) |
| Da Costa et al., 2016 | RRMS (20) 15F/5M | T CD3+ | 1,25(OH)2D3 (calcitriol) | 10 nM | 3 | without | PMA (20 ng/mL) + ionomycin (600 ng/mL) with brefeldin A (10 μg/ mL) | ELISA, FACS | ↑ IL-10,  ↓ IL-1β, IL-6, TNF α, IL 21, IFN γ, IL-17, IL-22 and GM-CSF | Fraction of : ↑ CD4 IL10+IL17+, Tr1 (Treg CD25+CD127-FoxP3-IL10+) ; Treg : CD4 IL10+IL17- ; CD25+CD127-FoxP3+ ; CD25+CD127-FoxP3+IL10+ ; CD8 IL10+IL17+, CD8 Treg (CD8 IL10+IL17-)  *↓* Th1 (CD4 IL17-IFN γ +), Th17 (CD4 IL10-IL17+), Th17 (CD4 IL17+IFN γ -), Th17.1 (CD4 IL17+IFN γ +), CD8 Tc1 (CD8 IL17-IFN γ +), CD8 Tc1* (CD8 IL17+IFN γ +), CD8 Tc17 (CD8 IL10-IL17+), CD8 Tc17 (CD8 IL17+IFN γ -) |
| Haas et al., 2016 | RRMS (10) | B CD19+ | 1α,25(OH)2D3 (Calcitriol) | 10-100ng/ml | 2 | without | 2 μg/ml CpG-ODN2006 or human CD40 mAb (5 μg/ml) plus IgM (12.5 μg/ml) | [3H] thymidine incorporation, ELISA | ↓ IL-6 | ↑ total B cells  B cells switched memory (CD20+CD27+IgD-)  naive B cells (CD20+CD27-IgD+) |
| Kickler et al., 2012 | RRMS (11) 9F/2M | T CD4+ | 1,25(OH)2D3 (calcitriol) | 100 nM | 5 | vehicle control (ethanol) | immobilized anti-CD3 or anti-CD3/CD46 + IL-2 (10 U/ml) | FACS, ELISA | ↑ CTLA4,  ↓ CD25, INF γ, CD46 if not associated with CD28+,  CD 28, IL10 | ↓ proliferation of CD4+ T cells  Treg (FoxP3+) |
| Lysandropoulos et al., 2011 | CIS (4) /RRMS (6) | T CD8+ | 1,25(OH)2D3 (calcitriol) | 100 nM | 7 | without | 1 μg/ml of a  pool of immunodominant 8–15 mer peptide EBV epitopes | FACS, ELISA | ↑ IL-5, TGF β,  ↓ IFN γ, TNF α,  IL-2, IL-4 and IL-10 | ↓ faction of IFN γ-secreting CD8+ T cells, faction of TNFα-secreting CD8+ T cells |
| Muris et al., 2016 | 30 RRMS 21F/9M | PBMC | 1,25(OH)2D3 (calcitriol) | 10 nM | 3 | without | Anti-CD3 stimulated PBMC | ELISA | ↑ IL-4, IL-5, LAP *,  ↓ IFN γ, IL-17, IL-22, GM-CSF, TNF α, IL- 10 |  |
| Peelen et al., 2015 | 8 RRMS 5F/3M | T CD4+ | 1,25(OH)2D3 (calcitriol) | 10 nM | 5 | without | anti-CD3 antibody + Brefeldin A | FACS | ↓ percentage inhibition of GM-CSF secretion  GM-CSF protein in cultur | percentage inhibition of GM-CSF+ cells  Fraction GMCSF cells |
| Sanseverino., et al 2014 | RRMS (10) | Mo-DCs | 1,25(OH)2D3 (calcitriol), 25(OH)D3 (calcidiol) | 10 nM, 1uM, | 6 | without | TLR4 agonist lipopolysaccharide (LPS) 10ng/ml | ELISA,  qRT-PCR | ↑ CCL2, ↑ CCL2 mRNA |  |
| Waschbisch., et al 2014 | RRMS (9) | Mo CD14 | 1,25(OH)2D3 (calcitriol) | 100 nM | 2 | Without | 10% of heat inactivated human AB serum, glutamine and antibiotics | FACS,  qRT-PCR | ↑ ILT3, ILT3mRNA, **↓** ILT4 |  |

**Supplementary Table 2: Effects of VitD on MS patients immune cells *in vitro*.**

The effects of Vitamin D are given as ↑ increased; ↓ decreased; not affected when not specified. † significant difference have been proved in female population against male population.

CCL : Chemokine (C–C motif) ligand, CLTA : cytotoxic T-lymphocyte-associated protein, ELISA : enzyme-linked immunosorbent assay, FACS : fluorescence-activated cell sorting analysis with cytometry, GM-CSF : Granulocyte-macrophage colony-stimulating factor; IDO : indoleamine 2 3-dioxygenase, IL: interleukin, ILT: Immunoglobulin-like transcript, INF :interferon, MMP :Matrix metallopeptidase, MoCD14 : Monocyte CD14, Mo-DCs : monocyte-derived dendritic cells, PBMC : peripheral blood mononuclear cells, qRT-PCR : quantitative real-time polymerase chain reaction, RRMS : relapsing remitting Multiples sclerosis, TGF :transforming growth factor, TNF : tumor necrosis factors, VDR : Vitamin D receptor

| **Reference** | **Study Model/Type** | **Vitamin D supplementation** | | **Effects of Vitamin D** | |
| --- | --- | --- | --- | --- | --- |
|  |  | **Isoform - Dose - Duration - Administration** | **Control** | **Method/Type of experiment** | **Observation** |
| **In Vitro** | | | | | |
| Garcion et al. 1996 | Primary brain micro vessels | 1,25(OH)2D3 - 10 ng/100g - 3-4 hours - i.p., only once | Vehicle (not mentioned) | $\gamma$-GT kit | = $\gamma$-GT activity (but 50-fold higher that in brain homogenate) |
|  | BBEC bovine cell line | 1,25(OH)2D3 - 1nM - 4, 18 hours - Addition to medium | Vehicle (not mentioned) | $\gamma$-GT kit | ↑ $\gamma$-GT activity (only when co-cultured with primary rat astrocytes) |
| Ito et al. 2011 | TM-BBB4 mouse cell line | N/A | N/A | Western Blot | VDR+ |
| Molinari et al. 2011 | HUVECs | 1,25(OH)2D3 - 0.01, 0.1, 1, 10 nM - 1 to 5 minutes - Addition to medium | EtOH | Griess Assay, DAF-FM | ↑ NO production (after 1 min treatment, completely reduced to 0 after 5 min) (VDR and NO synthase dependent) |
|  |  |  |  | Western Blot | ↑ p-eNOS, ↑ p-p38, ↑ p-Akt, ↑ p-ERK (VDR and NO synthase dependent) |
| Durk et al. 2012 | Sprague-Dawley rat brain capillaries | 1,25(OH)2D3 - 1, 10, 100 nM - 4 or 24 hours - Addition to medium | 0.1% EtOH | NBD-CSA assay | ↑ P-gP trasport activity |
|  |  |  |  | Western Blot | ↑ P-gP |
|  | RBE4 rat cell line | 1,25(OH)2D3 - 5, 10, 100 nM - 1, 3 days - Addition to medium | 0.1% EtOH | RT-qPCR | *↑ Vdr, ↑ Cyp24a1, = Mdr1a, ↑ Mdr1b* |
|  |  |  |  | Western Blot | ↑ P-gP |
|  |  |  |  | Rhodamine 6G accumulation | ↓ Rhodamine 6G accumulation |
|  |  |  |  | HiLyte Fluor 488 human Ameloid-β42 accumulation | ↓ HiLyte Fluor 488 human Amyloid-β1-42 accumulation |
|  | hCMEC/D3 human cell line | 1,25(OH)2D3 - 10, 100 nM - 1, 3 days - Addition to medium | 0.1% EtOH | RT-qPCR | ↑ *Vdr*, ↑ *Cyp24*, ↑ *Mdr1* |
|  |  |  |  | Western Blot | ↑ P-gP, = RAGE, = LRP-1 |
|  |  |  |  | Rhodamine 6G accumulation | ↓ Rhodamine 6G accumulation |
|  |  |  |  | HiLyte Fluor 488 human Ameloid-β42 accumulation | ↓ HiLyte Fluor 488 human Amyloid-β1-42 accumulation |
| Uberti et al. 2013 | HUVECs (H_2_O_2_ stimulation) | 1,25(OH)2D3 - 1 nM - 15 minutes - Addition to medium, prior H_2_O_2_ stimulation | EtOH | Griess Assay | ↑ NO production |
|  |  |  |  | MTT assay | ↑ Cell viability |
|  |  |  |  | Cytochrome C reduction | ↓ ROS production |
|  |  |  |  | Western Blot, Immunocytochemistry | ↑ p-Akt, ↑ p-ERK, ↑ Bectin-1 ↓ Bax, ↓ Caspase-3, ↓ Caspase-8, ↓ Caspase-9, ↓ Cytochrome C |
|  |  |  |  | Immunocytochemistry, TUNEL assay | ↓ Cell death |
|  |  |  |  | Measurement of MPTP, Membrane potential assay | Prevention of mitochondrial membrane potential loss and MPTP opening |
| Won et al., 2015 | bEnd.3 mouse cell line (Hypoxic condition) | 1,25(OH)2D3 - 0, 5, 20, 200 nM - 24 hours - Addition to medium, prior hypoxic condition | Untreated cells | FITC-Dextran fluorescein permeability test | ↓ Permeability |
|  |  |  |  | Millicell ERS-2 volt/ohm meter | ↑ TEER |
|  |  |  |  | Western Blot, Immunocytochemistry | ↑ ZO-1, ↑ Claudin-5, ↑ Occludin |
|  |  |  |  | MTT assay | ↑ Cell viability |
|  |  |  |  | MitoSOX Red dye | ↓ ROS production |
|  |  |  |  | Western Blot | ↓ MMP-9 |
|  |  |  |  | Immunocytochemistry | ↓ NF-kB activation (lower pNF-kB nuclear staining) |
| Guo et al. 2016 | bEnd.3 mouse cell line | 1,25(OH)2D3 - 1, 10, 100 nM - 60 hours - Addition to medium during hypoxic condition | 0.01% DMSO | Ameloid-β1–40 vectorial transport | ↑ Amyloid-β efflux |
|  |  |  |  | RT-qPCR, Western Blot | ↑ LRP-1, ↓ RAGE, ↑ VDR |
| Alam et al. 2017 | hCMEC/D3 human cell line | 1,25(OH)2D3 - 50, 100, 500 nM - 6, 24 hours - Addition to medium | EtOH | RT-qPCR | ↑ *RFC*, = *VDR*, ↑ *PCFT* |
|  |  |  |  | Western Blot, siRNA | ↑ RFC (VDR dipendent), ↑ PCFT |
|  | C57BL/129/LM/Bc mouse brain capillaries | 1,25(OH)2D3 - 100 nM - 4 hours - Addition to medium | EtOH | RT-qPCR | ↑ *Rfc*, = *Vdr* |
|  |  |  |  | Western Blot | ↑ RFC |
| Takahashi et al., 2017 | TY10 human cell line | 1,25(OH)2D3 - 1, 10, 100 nM - 24 hours - Addition to medium | Untreated cells | RT-qPCR, Western Blot, Immunocytochemistry | VDR+ (nuclear staining with VitD treatment; cytoplasmic without VitD treatment) |
|  |  |  |  | RT-qPCR | *Cyp24a1*+ (nuclear staining with VitD treatment; cytoplasmic without VitD treatment) |
|  |  |  |  | Western Blot | ↓ ICAM-1, ↑ Claudin-5, ↑ ZO-1 |
|  |  |  |  | Millicell electrical resistance apparatus | ↑ TEER |
|  |  | 1,25(OH)2D3 - 1, 10, 100 nM - 24 hours - Addition to medium, prior TNFa stimulation | Untreated cells | RT-qPCR, Western Blot, Immunocytochemistry | ↓ ICAM-1, ↑ Claudin-5, ↑ ZO-1 |
|  |  |  |  | Millicell electrical resistance apparatus | ↑ TEER |
|  |  |  |  | Immunocytochemistry | ↓ NFkB activation (lower pNF-kB nuclear staining) |
| Landel et al. 2018 | Primary brain endothelial cell culture from Wistar rats | N/A | N/A | RT-qPCR | *Cyp27a1+*, *Cyp27b1*+, *Cyp24a1*-, *Pdia3*+, *Vdr*+ (low) |
|  |  | 1,25(OH)2D3 - 20 nM - 12 hours - Addition to medium | EtOH | RT-qPCR | *= Cyp27a1*, = *Cyp27b1*, = *Vdr,* = *Cyp24a1,* ↓ *Pdia3* |
| Molinari et al. 2019 | HUVECs co-cultured with primary C57BL/6J mouse astrocytes | 25(OH)D3 - 100 nM - 15 to 1440 minutes - Addition to medium | Untreated cells | ELISA | Time-dependent ↑ in 25(OH)D3 permeability (stronger permeability in combination with lipoic acid) |
| **In Vivo** | | | | | |
| Garcion et al. 1996 | Sprague-Dawley rats | 1,25(OH)2D3 - 10 ng/100g - 2-3-4-8-24 hours - i.p., only once | Vehicle (not mentioned) | $\gamma$-GT kit | ↑ $\gamma$-GT cerebral activity |
| Ekici et al. 2009 | Wistar albino rats (tMCAO) | 1,25(OH)2D3 - 1 μg/kg - 8 days - i.p., every day, starting 8 days prior tMCAO | Sham-operated rats | Lipid peroxidation measurement | ↓ MDA levels (↓ Lipid peroxidation) (only in combination with DHA treatment) |
|  |  |  |  | GSH measurement | ↑ GSH activity (only in combination with DHA treatment) |
|  |  |  |  | SOD measurement | ↑ SOD activity (only in combination with DHA treatment) |
| Chow et al. 2011 | C57BL/6 mice fxr (-/-) | 1,25(OH)2D3 - 2.5 μg/kg - 8 days - i.p., every day | Sterile corn oil | RT-qPCR, Western Blot | ↑ P-gP, = VDR (FXR indipendent) |
|  |  |  |  | [3H] Digoxin analyses | ↑ Brain efflux |
| Ito et al. 2011 | C57BL/6J mice | 1,25(OH)2D3 - 1 µg/100µl/animal - 24 hours - i.p., once | 5% EtOH | Brain Efflux Index, ELISA | ↑ [125I] human and ↑ endogenous Amyloid-β1-40 elimination |
|  |  |  |  | Western Blot | VDR+ brain capillaries |
| Grishkan et al., 2013 | C57BL/6J mice (MOG_35–55_ EAE) | 1,25(OH)2D3 - 1.5 µg/kg - 15 days - Oral gavage, every day | 1% EtOH | Evans Blue dye extravasation | ↓ Permeability |
|  |  |  |  | Immunohistochemistry | ↓ IgG permeability |
| Durk et al. 2014 | C57BL/6 mice | 1,25(OH)2D3 - 2.5 µg/kg - 8 days - i.p., every second day | Sterile corn oil | Immunocytochemistry | P-gP+, VDR+ brain capillaries |
|  |  |  |  | Western Blot | ↑ Pg-P in the hippocampus |
|  |  |  |  | RT-qPCR | ↑ *Mdr1a*, ↑ *Cyp24a1* |
|  | C57BL/6 mice (Vitamin D-deficiet diet) | N/A | N/A | Enzyme immunoassay | ↓ Plasma 1,25(OH)2D3 |
|  |  |  |  | RT-qPCR, Western Blot | ↓ P-gP |
|  |  | 1,25(OH)2D3 - 2.5 µg/kg - 8 days - i.p., every second day | Sterile corn oil | Enzyme immunoassay | Rescued plasma 1,25(OH)2D3 levels |
|  |  |  |  | RT-qPCR, Western Blot | Rescued P-gP expression |
|  | human Ameloid-β transgenic mouse model (Tg2576) | 1,25(OH)2D3 - 2.5 µg/kg - 8 days - i.p., every second day | Sterile corn oil | RT-qPCR, Western Blot | ↑ P-gP, = BCRP, = LRP-1, = RAGE |
|  |  |  |  | ELISA | ↓ Human Amyloid-β1-40 and 1-42 accumulation |
|  | human Ameloid-β transgenic mouse model (TgCRND8) | 1,25(OH)2D3 - 2.5 µg/kg - 8 weeks - i.p., every third day | Sterile corn oil | RT-qPCR, Western Blot | ↑ P-gP, = LRP-1, = RAGE |
|  |  |  |  | ELISA | ↓ Human soluble and insoluble Amyloid-β1-40 and 1-42 accumulation |
|  |  |  |  | Immunocytochemistry | ↓ Cortical and hyppocampal plaque density, ↑ Cerebrovascular plaque density |
|  |  |  |  | Fear conditioning | ↑ Conditioned fear memory |
| Durk et al. 2015 | Sprague Dawley rats | 1,25(OH)2D3 - 4.8, 6.4 nmol/kg - 8 days - i.p., every 2 days | Sterile corn oil | Immunohistochemistry | P-gP+, VDR+ brain capillaries |
|  |  |  |  | RT-qPCR | ↑ *Cyp24a1*, ↑ *Mdr1a*, = *Mdr1*, = *Mdr4*, = *Bcrp* |
|  |  |  |  | Western Blot | ↑ P-gP, = MRP-1, = MRP-4, = BCRP |
|  |  |  |  | Equilibrium dialysis (Quinidine) | ↑ Brain efflux |
| Guo et al. 2016 | Kun Ming mice | 1,25(OH)2D3 - 500 nM - 0-60 minutes - Addition to medium | EtOH | [3H]-methotrexate uptake | ↑ Folate transport |
| Enkhjargal et al. 2017 | Sprague-Dawley rats | 1,25(OH)2D3 - 30, 60, 120 ng/kg - 24, 48, 72 hours - i.n., once | Not mentioned | Western Blot, Immunocytochemistry | ↑ Osteopontin (in astrocytes and endothelial cells) |
|  | Sprague-Dawley rats (SAH model) | 1,25(OH)2D3 - 30 ng/kg - 24, 72 hours - i.n., 24 hours before or 1 hour after SAH | Not mentioned | Neurobehavioral tests | ↓ Neurological deficit |
|  |  |  |  | Brain water content analysis | ↓ Brain edema |
|  |  |  |  | Evans Blue dye extravasation | ↓ Permeability |
|  |  |  |  | Western Blot, Immunocytochemistry | ↑ VDR, ↑ Osteopontin (in astrocytes) |
|  |  |  |  | RT-qPCR, siRNA | ↑ *Opn-a*, ↑ *Opn-c* (VDR dependent) |
|  |  |  |  | Western Blot, siRNA | Regulation of CD44 splice variants (VRD and OPN dependent) |
|  |  |  |  | Western Blot | ↑ P-gP glycosilation |
| Hajiluian et al. 2017 | Wistar rats (High fat diet) | Not mentioned - 500 IU/kg - 5 weeks - Oral gavage, every day | Migliol | Weighting | ↓ Body weight |
|  |  |  |  | ELISA | ↑ plasma 1,25(OH)2D3 levels |
|  |  |  |  | Morris water maze test | ↑ Cognitive function |
|  |  |  |  | Evans Blue dye extravasation | ↓ Permeability |
|  |  |  |  | Western Blot | ↓ NF-kB, ↑ BDNF |
| Evans et al. 2018 | C57BL/6 mice (tMCAO) | 1,25(OH)2D3 - 100 ng/kg - 5 days - i.p., every day, starting 5 days prior tMCAO | Sterile water, propylene glycol, EtOH (5:4:1 ratio) | Thionin staining | ↓ Brain infarct volume |
|  |  |  |  | Neurobehavioral tests | = Neurological deficit |
| Alam et al. 2019 | C57BL/129/LM/Bc mouse (WT or Folr1 KO) | 1,25(OH)2D3 - 2.5 μg/kg - 8 to 16 days - i.p., every day | Sterile corn oil | Body weighting | ↓ Body weight |
|  |  |  |  |  | ↑ Folate uptake (↑ Transport activity of RFC) (in Folr1 KO mice only) |
|  | C57BL/129/LM/Bc mouse isolated brain capillaries (WT or Folr1 KO) | 1,25(OH)2D3 - 2.5 μg/kg - 8 days - i.p., every day | Sterile corn oil | RT-qPCR | *↑ Rfc, ↑ P-gP, ↑ Pcft, = Oat3, = Mrp1, = Mrp2* |
| Sadeghian et al. 2019 | Sprague Dawley rats (tMCAO) | 1,25(OH)2D3 - 12 μg/kg - 24 hours - i.p., once, 4 hours after tMCAO | EtOH | TCC staining | ↓ Infarct size |
|  |  |  |  | Brain water content analysis | ↓ Brain edema |
|  |  |  |  | Evans Blue dye extravasation | ↓ Permeability |
|  |  |  |  | Assay kits | ↓ MDA concentration, ↑ SOD, ↑ CAT, ↑ GPX activities |
|  | Sprague Dawley rats (tMCAO) | 1,25(OH)2D3 - 2 μg/kg - 5-7 days - i.p., every day | EtOH in saline solution | Neurological deficit score (NDS) | ↓ Neurological deficit |
|  |  |  |  | H&E staining | ↓ Pathological changes |
|  |  |  |  | Tunel staining | ↓ Apoptosis |
|  |  |  |  | Immunocytochemistry | ↑ BDNF |
| Sayeed et al. 2019 | Wistar rats (tMCAO, Vitamin D-deficiet diet) | N/A | N/A | ELISA | ↓ Serum 1,25(OH)2D3 levels |
|  |  |  |  | Nissl staining | ↑ Brain infarct volume |
|  |  |  |  | Immunohistochemistry, Western Blot | ↑ IgG permeability, ↑ MMP-9, ↓ Claudin-5, ↓ Occludin, ↓ β-Catenin, ↑ VDR, ↑ GLUT-1, ↑ Osteopontin |
| de Oliveira et al., 2020 | C57BL/6J mice (MOG_35–55_ EAE) | 1,25(OH)2D3 - 5 µg/kg - 8 days - i.p., every day, starting 24 hours after immunization | 15% EtOH | NaFlu permeability test | ↓ Permeability |
|  |  |  |  | RT-qPCR | ↑ *ZO-1* |
| Peng et al. 2020 | Sprague Dawley rats (Human Amyloid-β-40 injected) | 1,25(OH)2D3 - 1 μg/kg - 4 days - i.p., every day, starting 4 days prior Amyloid-β-40 injection | Sterile corn oil | Western Blot | ↑ P-gP, = Neprilysin, = Lrp1, = Mrp1 |
|  |  |  |  | ELISA | ↑ Human Amyloid-β-40 efflux |
| Yang et al. 2021 | Sprague–Dawley rats (TBI model) | Cholecalciferol - 1, 2, 5 μg/kg - 7 days - i.p., every day, starting 7 days prior TBI | Almond oil | Enzyme-linked immunosorbent assay | ↑ Serum 25(OH)D3 |
|  |  |  |  | Weighing wet and dry weight | ↓ Brain edema |
|  |  |  |  | Motor, sensory, reflex, and balance tests | ↓ Neurological deficit |
|  |  |  |  | MWM test | ↓ Cognitive impairment |
|  |  |  |  | Evans Blue dye extravasation | ↓ Permeability |
|  |  |  |  | Western Blot | ↑ ZO-1, ↑ Occludin |
|  |  |  |  | RT-qPCR | ↓ *TNF-α, ↓ IL-6, ↓ IL-1β* |
| **MS patients/ derived samples** | | | | | |
| Kimball et al. 2007 | RRMS or SPMS patients | Cholecalciferol - From 28’000 to 280’000 - 28 weeks - Oral administration, increasing dosage through the 28 weeks of treatment | Placebo | MRI | ↓ Numbers of new Gd-enhancing lesions |
| Mosayebi et al. 2011 | MS patients | Cholecalciferol - 300’000 IU - 6 month - i.m., every month | Placebo | MRI | = Numbers of new Gd-enhancing lesions |
| Soilu-Hänninen et al. 2012 | RRMS patients with at least 1-month IFNβ-1b treatment) | Cholecalciferol - 20’000 IU - 12 month - i.m., every week | Placebo | MRI | ↓ Numbers of new Gd-enhancing lesions |
| Dehghani et al., 2013 | HUVECs (MS serum treatment) | 1,25(OH)2D3 - 100 nM - 24 hours - Addition to medium, prior MS patient's serum addition | Untreated cells | MTS assay | ↑ Cell viability |
|  |  |  |  | ELISA | ↓ Apoptosis |
| Takahashi et al., 2017 | TY10 human cell line (MS serum treatment) | 1,25(OH)2D3 - 1, 10, 100 nM - 24 hours - Addition to medium, prior MS patient's serum addition | Untreated cells | Western Blot | ↓ VCAM-1, ↓ NFkB, ↑ Claudin-5, ↑ ZO-1 |
| Hupperts et al., 2019 | RRMS patients with INFβ-1a treatment | Cholecalciferol - 14'007 IU - 48 weeks - Oral administration, every day | Placebo | MRI | ↓ Numbers of new Gd-enhancing lesions, ↓ New/enlarging T2 lesions |

**Supplementary Table 3. The effects of vitamin D on the BBB.**

The effects of Vitamin D are given as ↑ increased; ↓ decreased; = not affected. Changes in protein and mRNA expression levels are indicated respectively with normal and *italic* text. Combined data on protein and mRNA are written with normal text.

| **Reference** | **Study Model/Type** | **Vitamin D supplementation** | | **Effects of Vitamin D** | |
| --- | --- | --- | --- | --- | --- |
|  |  | **Isoform - Dose - Duration - Administration** | **Control** | **Method/Type of experiment** | **Observation** |
| **In Vitro** | | | | | |
| Nissou et al. 2014 | Human brain pericytes | N/A | N/A | Western Blot | VDR+ |
|  |  | 1,25(OH)2D3 - 10 nM - 24 hours - Addition to medium | Ethanol | RT-qPCR | ↑ *Cyp24a1* |
|  |  |  |  | Microarray, RT-qPCR | *Cyp27b1*-, ↑ *Cyp24a1*, ↑ *Vcam-1*, ↑ *Eftud1*, ↑ *Thbd*, ↑ *Osr2*, ↑ *Klf4*, ↑ *Serinc2*, ↑ *Tnfaip6,* ↑ *Gata6,* ↑ *Kcjn2*, ↑ *Cables1*, ↑ *Col16a1*, ↑ *Elfn1*, ↑ *Chrdl1*, ↑ *Cd248,* ↑ *Serpine2*, ↑ *Igfbp5*, ↑ *Rarres1*, ↑ *Lrrn3,* ↑ *Epas1*, ↓ *Ccl2*, ↓ *Fabp4,* ↓ *Tnfs4/Ox40l*, ↓ *Ak5*, ↓ *Adamtsl1* |
| El-Atifi et al. 2015 | Human brain pericytes  TNF$\alpha$ and IFN$\gamma$ treatment | N/A | N/A | RT-qPCR | ↑ *Cypr1*, ↑ *Vdr* |
| Takahashi et al. 2017 | Human brain pericyte cell line (HBPCT) | N/A | N/A | RT-qPCR, Western Blot | VDR+ |
| Jamali et al. 2019 | Primary retinal pericytes from WT C57BL/6J mice and (VDR)-deficient mice (B6.129S4-Vdr^tm1Mbd^/J) | N/A | N/A | FACS | ↑ NG2 and ↑ Smooth Muscle Actin (in VDR KO cells) |
|  |  | 1,25(OH)2D3 - 10 μM - 48 hours - Addition to medium | Ethanol | Counting, Click‐It‐EdU Alexa Flour 488 assay, TUNEL assay, VEGF trap (sFLT‐1) | ↓ Cell count (VDR dependent), ↓ DNA synthesis (VDR dependent), no change in apoptosis rate, ↑ enhanced G1 arrest (VEGF dependent) |
|  |  |  |  | Scratch wound assay | ↓ Wound closure |
|  |  |  |  | Transwell migration assay, VEGF trap (sFLT‐1) | ↓ Migration (VDR independent, VEGF dependent) |
|  |  |  |  | Cell adhesion assay | ↑ Adhesion to ECM (in both WT and Vdr-deficient cells) |
|  |  |  |  | FACS | ↑ $\alpha$4 integrin, ↑ $\alpha$5$\beta$1 integrin |
|  |  |  |  | ELISA | ↑ VEGF production |
|  |  |  |  | Migration assay | ↑ Migration of endothelial cells (upon pericytes conditioned treatment) |
|  |  |  |  | Amnis imaging flow cytometry analysis | ↑ VEGFR2-PDGF$\beta$R heterodimer |
|  |  | 1,25(OH)2D3 - 10 μM - 24 hours - Addition to medium | Ethanol | Western Blot, RT-qPCR, VEGF trap (sFLT‐1) | ↑ p38 activation, ↑ ERK activation, ↓ AKT activation, ↑ VDR (VEGF dependent) |
| **In Vivo** | | | | | |
| Garcion et al. 1996 | Primary brain pericytes from Sprague-Dawley rats | 1,25(OH)2D3 - 1nM - 18 hours - Addition to medium | Vehicle (not mentioned) | $\gamma$-GT kit | ↑ $\gamma$-GT activity |
| Chung et al. 2009 | C57BL/6j mice  VDR (-/-)  TRAMP tumours implantation | N/A | N/A | Immunohistochemistry | ↓ Pericytes coverage on TRAMP-2 tumor’s vessels |
|  |  |  |  | ELISA | VEGF secretion ↑ |
|  |  |  |  | Western Blot | PDGF-Rβ ↓ |
| Jamali et al. 2017 | C57BL/6j mice  (Vdr)-deficient (B6.129S4-Vdr^tm1Mbd^/J) | N/A | N/A | Retinal mount, Immunocytochemistry | No change in angiogenic sprouting and retina vascular coverage (P5)  No change in number of arteries, branches and point branches (P7, P21)  ↓ Endothelial/pericyte cell ratio (P42) |
|  |  |  |  | Body weighting | No change in body weight (P17) |
|  | C57BL/6j mice  (Vdr)-deficient (B6.129S4-Vdr^tm1Mbd^/J)  Oxygen induced ischemic retinopathy model (OIR) | N/A | N/A | Retinal mount, Immunocytochemistry | No change in neovascularization and area of vessel obliteration (P17) |
|  |  | 1,25(OH)2D3 - 2.5 µg/kg - 5 days - i.p. every day | Saline solution | Retinal mount, Immunocytochemistry | ↓ Neovascularization (VDR dependent) (P17), no change in area of vessel obliteration (P17) |
|  |  |  |  | Body weighting | No change in body weight (VDR dependent) (P17) |

**Supplementary Table 4. The effects of vitamin D on pericytes.**

The effects of Vitamin D are given as ↑ increased; ↓ decreased; = not affected. Changes in protein and mRNA expression levels are indicated respectively with normal and *italic* text. Combined data on protein and mRNA are written with normal text.

| **Reference** | **Study Model/Type** | **Vitamin D supplementation** | | **Effects of Vitamin D** | | |
| --- | --- | --- | --- | --- | --- | --- |
|  |  | **Isoform - Dose - Duration - Administration** | **Control** | **Method/Type of experiment** | **Observation** | |
| **In Vitro** | | | | | | |
| Neveu, et al. 1994 | Primary astrocyte culture, C6 glioma cells | 1,25(OH)2D3 - 10 nM - 24 hours - Addition to medium | Ethanol | RNAse protection assay | ↑ *NGF* (astrocytes), ↓ *NT-4* (C6 glioma) |  |
| Neveu, et al. 1994 | Primary rat brain astrocytes, C6 glioma cells | 1,25(OH)2D3 - 10 nM - 6 , 48 hours - Addition to medium | Untreated cells  (N/A) | Northern blotting | ↑ *Ngf*, ↑ *App* (astrocytes and glioma cells) |  |
|  |  |  |  | ELISA | ↑ NGF, ↑ VDR (astrocytes and glioma cells) |  |
| Naveilhan et al., 1996 | Primary astrocyte culture, C6 glioma cells | N/A | N/A | Northern blotting | *trkA-*, *trkB+ & trkC+* (astrocytes and glioma cells) |  |
|  |  | 1,25(OH)2D3 - 0.1 to 10 nM - 12-48 hours - Addition to medium | 10% ethanol in propylene glycol | Northern blotting | *↑ P75^NTR^, ↑ Vdr* |  |
| Garcion et al. 1996 | Primary brain astrocytes from Sprague-Dawley rats | 1,25(OH)2D3 - 1nM - 4, 18 hours - Addition to medium | Vehicle (not mentioned) | $\gamma$-GT kit | ↑ $\gamma$-GT activity | |
| Hahn et al., 1997 | RC7 immortalized cerebral astrocytes and/or with corticosterone | 1,25(OH)2D3 - 1 µM - 38 hours - Addition to medium | Vehicle (not mentioned) | Two-sided enzyme assay | ↑ NGF | |
| Baas et al. 2000 | Rat CG-4 cell line | 1,25(OH)2D3 - 100 nM - 24 hours - Addition to medium | Vehicle (not mentioned) | Northern blot | *Vdr*+ type-2 astrocytes | |
| Smolders et al. 2013 | Astroglioma cell lines (U343, U373) | 1,25(OH)2D3 - 100, 1000 nM - 48 hours - Addition to medium | EtOH | RT-qPCR | *= Cyp27b1, ↑ Cyp24a1, ↑ Vdr* | |
| Shirazi et al. 2015 | Neural stem cells (NSCs) from C57BL/6J mice | 1,25(OH)2D3 - from 10 pM to 10nM - 14 days - Addition to medium | None | Immunocytochemistry | ↑ GFAP+ cell differentiation (low) (astrocytes) | |
| Jiao et al. 2017 |  |  |  |  |  | |
| Landel et al., 2018 | Primary astrocytes culture from Wistar rats | N/A | N/A | RT-qPCR | *Cyp27a1+*, *Cyp27b1*-, *Cyp24a1*-, *Pdia3*+, *VDR*+ (high) | |
|  |  | 1,25(OH)2D3 - 20 nM - 12 hours - Addition to medium | EtOH | RT-qPCR | *= Cyp27a1*, = *Cyp27b1*, *= Vdr,* ↑ *Cyp24a1, = Pdia3* | |
| Ferronato et al. 2018 | Glioblastoma cells (U251, T98G); Human primary astrocytes | 1,25(OH)2D3 or EM-1 (analogue of 1,25(OH)2D3) - 100 nM - 17 hours - Addition to medium | Isopropanol | Cell Proliferation assay | ↑ % cells in G0/G1 phase | |
|  |  |  |  | Western blot | ↑ p21, ↑ p27, ↑ p57, ↓ cyclin | |
|  |  |  |  | Cell viability assay | ↓ Primary astrocytes Viability % (1,25(OH)2D3), ↑ Viability % (EM-1) | |
|  |  |  |  | Wound healing assay | ↓ Migration (T98G and U251) | |
|  |  |  |  | Transwell invasion assays | ↓ Cell invasion (EM-1), ↑ 1,25(OH)2D3 (relative to EM-1) (T98G) | |
|  |  |  |  | Gelatin Zymography assay | ↓ MMP9 activity (T98G) | |
| Molinari et al. 2019 | Primary astrocytes from C57BL/6J mice (H_2_O_2_ stimulation) | 1,25(OH)2D3 - 100 nM - 15 to 1440 min - Addition in medium in combination or not with LA | Untreated cells | Cell viability assay | ↑ Viability (alone and combined with LA) | |
|  |  |  |  | ROS Production assay & Membrane potential assessment | ↓ ROS production (alone and combined with LA), ↑ Mitochondrial activity (alone and combined with LA) | |
|  |  |  |  | ELISA kit -p53 transcription factor assay kit | ↓ p53 activity (alone and combined with LA) | |
|  |  |  |  | APP Quantification | ↓ APP | |
|  |  |  |  | ERK and Akt Activation Assay | ↑ p-EKR activity, ↑ p-Akt activity (alone and combined with LA) | |
|  |  |  |  | Iron Quantification Assay | ↓ Iron uptake | |
|  |  |  |  | Western blot | ↓ SOD3, ↓ iNOS, ↑ p-Akt, ↑ Cytochrome C | |
| Clarke et al. 2020 | Primary human microglia from patients undergoing surgery for intractable epilepsy | 1,25(OH)2D3 - 100 nM - 6 days - Addition to medium | Vehicle (not mentioned) | RT-qPCR, FACS | = MerTK | |
| **In Vivo** | | | | | | |
| Garcion et al. 1997 | Lewis rats (GPSC EAE) | 1,25(OH)2D3 - 5 and 1 µg/kg - 23 days - i.p., starting with 5 µg/kg on d11 and d13 post immunisation and continuing with 1 µg/kg on d19, d21, d23 | Propylene glycol and disodium phosphate | Immunohistochemistry, In-situ hybridisation | ↓ GFAP+iNOS+ astrocytes in the cerebellum, brainstem and spinal cord | |
| Spach et al. 2004 | B10.PL(73NS)/Sn mice (MBP EAE) | 1,25(OH)2D3 - 2 μg/ml - 6 hours - i.p. | Soybean oil | RT-qPCR | *↓ GFAP; ↑ Calcium/calmodulin-dependent protein kinase IIδ* | |
| Hua et.al, 2012 | Male Sprague–Dawley rat (TBI model) | 1,25(OH)2D3 - 1, 2.5 or 5 μg/kg – 1h post-injury – i.p, only once, given with 16 mg/kg Progesterone (PROG) | Lesion (CCl)-PROG | Immunohistochemistry | = % GFAP+ cells, ↑ Size of processes and cell body (significant for 1 μg/kg) | |
|  |  |  | Sham + Vehicle (22.5% 2-hydroxyropyl-β-cyclodextran (HBC)+ Sterile water) |  | = % GFAP + cells, ↑ Size of processes and cell body | |
| Nystad et al. 2014 | C57BL/6J mice (Cuprizone Model) | 1,25(OH)2D3 - 0.2 ug/mouse - 4 weeks - i.p., twice weekly, starting 5 weeks after cuprizone treatment | Vehicle (not mentioned) | Immunohistochemistry | ↑ GFAP+ astrocytes (astrocytosis) | |
| Tang et al. 2015 | Sprague-Dawley rats (TBI model) | N/A | N/A | Immunocytochemistry | GFAP+ cells arranged around lesion site | |
|  |  | 1,25(OH)2D3 - 1 mg/kg - 1, 6 hours post-injury - i.p., once | PBS | Western Blot | ↓ GFAP, ↓ TLR4 | |
| Malinic & Dozudic 2017 | Mongolian Gerbils (Transient global cerebral ischemia) | Not mentioned - Not mentioned - 7 days - Prior to ischemia (method not mentioned) | Untreated mice | Western Blot | ↑ GFAP | |
| Calvello et al. 2017 | C57BL/6N mice (MPTP model of Parkinson’s disease) | 1,25(OH)2D3 - 1 μg/kg - 10 days - Intragastric gavage, starting 3 days before MPTP injection | Saline solution | Immunohistochemistry | ↑ GFAP+ cells | |
|  |  |  |  | Western Blot | ↓ GFAP | |
| Gomez-Pinedo et al. 2019 | Wistar rats (Surgical lesions to the corpus callosum and lysolecithin injection) | Cholecalciferol - 5’000 IU/kg - 30 days - not mentioned, every day, 15 days prior and after to lysolecithin injection | None | Immunohistochemistry | Subventricular zone: = GFAP+ cells  Corpus callosum (lesion site): ↑ GFAP+ cells | |
| Zhang et al. 2020 | C57BL/6J (MOG_35-55_  EAE) | Paricalcitriol (Vitamin D2 analogue) - 0.25, 0.5 μg/kg - i.p., every 2 days from d2 to d28 | Untreated mice | Western Blot, Immunohistochemistry | ↓ GFAP+ astrocytes, ↓ Iba1+ microglia | |
|  |  |  |  | Immunohistochemistry | ↓ F4/80, ↓ iNOS, ↓ COX2 | |
| **MS patients / derived samples** | | | | | | |
| Smolders et al. 2013 | Post-mortem brain tissue of MS and control patients (NAWM) | N/A | N/A | Immunohistochemistry | GFAP+VDR+ cells (astrocytes) (nuclear, but also cytoplasmic, unlike controls)  GFAP+CYP24A1+ cells (astrocytes) | |
|  | Primary human astrocyte culture from post-mortem MS-brain tissue | 1,25(OH)2D3 - 100-1000 nM - 48 hours - Addition to medium | EtOH | Immunocytochemistry | ↑ CYP24A1 | |
| de la Fuente et al. 2015 | Post-mortem brain tissue of MS and control patients (NAWM, active and chronic lesions, PPWM) | N/A | N/A | Immunohistochemistry | GFAP+VDR+ cells (astrocytes)  ↑ VDR+ cell in active lesions, ↑ nuclear VDR staining in active lesions, = cytoplasmic VDR staining in all lesions | |

**Supplementary Table 5. The effects of vitamin D on astrocytes.**

The effects of Vitamin D are given as ↑ increased; ↓ decreased; = not affected. Changes in protein and mRNA expression levels are indicated respectively with normal and *italic* text. Combined data on protein and mRNA are written with normal text.

| **Reference** | **Study Model/Type** | **Vitamin D supplementation** | | **Effects of Vitamin D** | |
| --- | --- | --- | --- | --- | --- |
|  |  | **Isoform - Dose - Duration - Administration** | **Control** | **Method/Type of experiment** | **Observation** |
| **In Vitro** | | | | | |
| Baas et al. 2000 | Secondary Oligodentrocytes cultures from Sprague-Dawley rat | 1,25(OH)2D3 - 100 nM - 24 hours - addition to medium | Vehicle (not mentioned) | In situ hybridization | *Vdr*+ (cytoplasmic localization) |
|  |  |  |  | Immunocytochemistry | ↑ VDR+ cells (nuclear and cytoplasmic localization) |
|  |  |  |  | Northern blot | ↑ *Vdr,* ↑ *Ngf,* ↑ *p75^NTR^, = Mbp, = Plp* |
|  | Rat CG-4 cell line | 1,25(OH)2D3 - 100 nM - 24 hours - addition to medium | Vehicle (not mentioned) | Northern blot | *VDR*+ O-2A progenitors, *VDR*+ Oligodentrocytes |
| Chabas et al. 2013 | Schwann cells and mixed culture of neurons and schwann cells from dorsal root ganglia of Sprague Dawley rats | 1,25(OH)2D3 - 500 nM - 24 hours - Addition to medium | Vehicle (not mentioned) | Microarray, qPCR  Analysis for significant gene ontology clusters using DAVID Bioinformatics Resources | *↑ 89 and ↓ 42 genes expression, Regulation of the expression of genes involved in axogenesis and myelination (Acsl4, Arhgef19, Il33, Pank1, Relt and Spp1 in both cultures),*  Altered functions in DRG/Schwann cell cultures: *mRNA processing, ribonucleotide binding, neurological system process, regulation of apoptosis, muscle development*  Altered functions in Schwann cell only cultures: *anion binding, steroid metabolic process, response to hormone stimulus, inflammatory response, cell cycle*  Commonly activated pathways: *regulation of axogenesis, regulation of cytoskeleton, regulation of blood pressure, regulation of cell migration, regulation of transcription (some genes in myelination)* |
| Shirazi et al. 2015 | Neural stem cells (NSCs) from C57BL/6J mice | 1,25(OH)2D3 - from 10 pM to 10 nM - 5 days - addition to medium | None | RT-qPCR | *Vdr+,* ↑ *Vdr* (concentration dependent manner), ↑ *Nt-3*, ↑ *Bdnf*, ↑ *Cntf*, ↑ *Gdnf* |
|  |  |  |  | Hemocytometer counting, BrdU assay | ↑ Number of cells, ↑ Cell size, ↑ Neurosphere formation |
|  |  | 1,25(OH)2D3 - from 10 pM to 10 nM - 14 days - addition to medium | None | Widefield microscopy | ↑ Mature/differentiated morphology |
|  |  |  |  | Immunocytochemistry | ↑ GalC+ cell differentiation (oligodendrocytes), = Nestin+ undifferentiation (undifferentiated NSCs) |
| de la Fuente et al. 2015 | Oligodendrocytes and oligodendrocytes progenitor cells (OPCs) from Sprague-Dawley rats | N/A | N/A | Immunocytochemistry, Western Blot, Co-IP, Proximity ligand assay | VDR+, RXR$\gamma$+, VDR+RXR$\gamma$+, (= nuclear and cytoplasmic amount) (both OLGs and OPCs)  VDR antagonists or VDR siRNA: *↓* VDR, ↑ OPC Proliferation (Ki67+ cells), *↓* Differentiation (MBP+ cells), *↓* MBP production, = Cell death  VDR antagonist + RXR agonist: *↓* MBP+ cells |
|  |  | 1,25(OH)2D3 - 10-50-100 nM - 24 hours - addition to medium | DMSO | Immunocytochemistry, Western blot | = OPCs Proliferation, = Differentiation, = Survival (normal FBS in medium)  ↓ OPCs Proliferation (Ki67+ cells), ↑ Differentiation (MBP+ cells), ↑ MBP production, = Survival (charcoal-stripped FBS in medium) (VDR dependent) |
| Landel et al. 2018 | Wistar rats derived Oligodentrocytes | N/A | N/A | RT-qPCR | *Cyp27a1+*, *Cyp27b1*-, *Cyp24a1*-, *Pdia3*+, *VDR*+ (low) |
| Mengozzi et al. 2020 | Rat CG-4 cell line | 1,25(OH)2D2 or 1,25(OH)2D3 - 100 nM - 24 or 72 hours hours - addition to medium | 96% EtOH | RT-qPCR, Microarray | ↑ *Vdr* (for both 24- and 72-hours incubation)  Vitamin D3 influenced 1272 genes and vitamin D2 574 at 24 hours (D3 > D2)  *↓ Ontology category gene expression, ↓ Transcription factor genes (Sox4, Tfeb), ↓ Notch pathway genes (Jag1, Dll3, Psen1), ↑ Ras pathway genes (RRas2, Ngf, Tgfb2)* |
| **In Vivo** | | | | | |
| Baas et al. 2000 | Wistar rats | N/A | N/A | Immunohistochemistry | VDR+ (30-50% in the striatum, 10% in the corpus callosum or spinal cord) |
| Nashold et al. 2000 | B10.PL(73NS)/Sn mice  MBP EAE | 1,25(OH)2D3 - 100 ng (injection) + 50 ng (diet) - 72 hours - i.p., every day, starting at moderate EAE severity + Additional food supply every day | Soybean oil (injection), 1,25(OH)2D3 devoid diet | Immunohistochemistry | ↓ Demyelination, ↑ Myelination |
| Goudarzvand et al. 2010 | Sprague Dawley rats  Ethidium bromide model | 1,25(OH)2D3 - 5 μg/kg - 2-7-28 days - i.p., every day, starting post lesion | Soybean oil | Immunohistochemistry | = Demyelination, = Myelination (2- and 7-days post lesion)  ↓ Demyelination, ↑ Myelination (28 days post lesion) |
|  |  |  |  | Western Blot | = Caspase-3 activation (2 days), ↓ Caspase-3 activation (7- and 28-days)  = MBP (7 days), ↑ MBP (2- and 28-days) |
| Wergeland et al. 2011 | C57BL/6J mice  Cuprizone Model | Not mentioned (Vitamin D3) - 50, 500, 6200, 12’500 IU/kg - 10 weeks - 2 weeks before cuprizone addition, diet supplement | Cuprizone untreated mice | LC/MS-detector, Body weighting | ↑ Serum 25(OH)D3 (dose dependent), ↑ Serum calcium (dose independent), = Body weight |
|  |  |  |  | Immunohistochemistry, LFB/PLP scores | ↓ Demyelination, = Number of NOGO-A+ oligodendrocytes, = Remyelination (high vs low Vitamin D diet) |
| Chabas et al. 2013 | Sprague-Dawley rats with peroneal nerve injury | Cholecalciferol (Vitamin D3) or ergocalciferol (Vitamin D2) - 100 or 500 IU/kg/day - 3 months - Oral gavage, every week | Triglicerides | Peroneal functional index (PFI), | ↑ Locomotor recovery (after 3 months in 500 IU/kg/day treated animals) |
|  |  |  |  | Maximum relaxation rate (MRR), Tetanus threshold | ↑ Muscle mechanical properties (after 3 months in 500 IU/kg/day treated animals) |
|  |  |  |  | Ventilation response, Metabosensitive afferent activity | ↑ Ventilation and metabosensitive afferent responses after active muscle electrical stimulation or intramuscular capsaicin injection |
|  |  |  |  | Tibialis anterior muscle weighting | ↓ Muscular atrophy |
|  |  |  |  | Immunohistochemistry (G-ratio) | ↑ Axon numbers (in the proximal area), ↑ axon diameter, ↑ Myelination (in the proximal and distal area) |
|  |  |  |  | ELISA | = Calcium levels, ↑ Serum 25(OH)D3 |
| Nystad et al. 2014 | C57BL/6J mice  Cuprizone Model | 1,25(OH)2D3 - 0.2 ug/mouse - 4 weeks - i.p., twice weekly, starting 5 weeks after cuprizone treatment | Vehicle (not mentioned) | Immunohistochemistry, LFB scores | ↓ Demyelination, ↑ Remyelination, ↑ Number of NOGO-A+ oligodendrocytes |
|  |  |  |  | LC/MS-detector | = Serum calcium, ↓ Serum 25(OH)D3, ↓ Serum 24,25(OH)2D3 |
|  |  |  |  | Body weighting, RotaRod test | = Body weight, = Motor performance |
| de la Fuente et al. 2015 | Sprague-Dawley rats  Ethidium bromide model | N/A | N/A | Immunohistochemistry | APC+VDR+ cells, Olig2+VDR+ cells (nuclear staining) (21 day post lesion (dpl))  ↓ Olig2+VDR+ cells (5 dpl), ↓ Olig2+VDR+ cells (14 and 21 dpl),  ↑ Olig2+APC-VDR+ cells (OPCs and immature OLGs) (5 dpl), ↑ APC+VDR+ cells (14 and 21 dpl) |
|  | Cerebellar slices culture from Sprague-Dawley rats  Lysolecithin-induced demyelination | VDR antagonist (ZK159222) | DMSO | Immunohistochemistry | ↓ Myelination (Area MBP-NHF/NHF) (wo Lysolecithin)  ↓ Remyelination (ArearMBP-NHF/NHF) (with Lysolecithin) |
| Montava et al. 2015 | New Zealand white rabbits with surgical lesion to the facial nerve | Cholecalciferol - 200 IU/kg - 12 week - Oral administration, every day, starting 1 post-surgery | Triglycerides | Facial functional index (FFI) | ↓ Facial paralysis (from week 6 on) |
|  |  |  |  | Immunohistochemistry | = Inner diameter of myelin sheath, ↑ Myelination (G ratio) |
| Mashayekhi et al. 2016 | Balb/c mice  Cuprizone Model | Not mentioned (Vitamin D3) - 5 μg/kg - 6 weeks - i.p., every day, starting 5 weeks after cuprizone treatment | Olive oil | Bio-Rad protein assay, Western Blot | = Total brain protein amount, ↑ CNPase, ↑ MOG |
| Shirazi et al. 2017 | C57BL/6J mice  MOG_35–55_ EAE | 1,25(OH)2D3 - 0.1 μg - 15 days - i.p., every second day from day 15 to 30 p.i. | 0.1% EtOH | Immunohistochemistry | ↓ Demyelination, ↑ NG2 (OPCs), ↑ GalC (oligodendrocytes), ↑ Nestin (neural stem cells), ↑ MBP (myelin sheath), |
| Nystad et al. 2018 | C57BL/6J mice  Cuprizone model | Cholecalciferol - 50-500-6200-12500 IU/kg - 9 weeks - Addition to food, every day, starting 2 weeks before cuprizone treatment and stopping at the end of cuprizone treatment (demyelination phase) | Chow w/o Cholecalciferol | Immunohistochemistry | ↓ Axonal loss (↑ NFL area) (dose dependent), = Axonal damage (SMI-32 area), = Axonal transection (APP density) |
|  |  | 1,25(OH)2D3 - 2 μg - 5 weeks - i.p., twice weekly starting 2 weeks before the end of cuprizone treatment and stopping 3 weeks after cuprizone treatment 3 weeks (remyelination phase) | Placebo | Immunohistochemistry | = Axonal loss (↑ NFL area), = Axonal damage (SMI-32 area), = Axonal transection (APP density) |
| Gomez-Pinedo et al. 2019 | Wistar rats  Surgical lesions to the corpus callosum and lysolecithin injection | Cholecalciferol - 5,000 IU/kg - 30 days - not mentioned, every day, 15 days prior and after to lysolecithin injection | None | Immunohistochemistry | Subventricular zone: ↑ BrdU+ cells, ↑ DCX+ cells  Corpus callosum (lesion site): ↑ BrdU+ cells, ↑ DCX+ cells, ↑ Olig2+ cells, *↓* NeuN+ (Cas3+), ↑ MBP expression, = LRP2 expression, ↑ PLP expression, ↑ NG2+ cells, *↓* Iba1+ cells |
|  |  |  |  | Weighting | = Body weight |
| **MS patients / derived samples** | | | | | |
| Smolders et al. 2013 | Postmortem brain tissue of MS and control patients (NAWM) | N/A | N/A | Immunohistochemistry | VDR+ cells (both MS and control patients), many positive nuclei lined up in linear conformations, suggestive of oligodendrocytes. |
| de la Fuente et al. 2015 | Post-mortem brain tissue of MS and control patients (NAWM, active and chronic lesions, PPWM) | N/A | N/A | Immunohistochemistry | GFAP+VDR+ cells (astrocytes)  ↑ VDR+ cell in active lesions, the only remyelinated lesion identified was full of VDR+ cells, ↑ nuclear VDR staining in active lesions, = cytoplasmic VDR staining in all lesions |

**Supplementary Table 6. The effects of vitamin D on oligodendrocytes.**

The effects of Vitamin D are given as ↑ increased; ↓ decreased; = not affected. Changes in protein and mRNA expression levels are indicated respectively with normal and *italic* text. Combined data on protein and mRNA are written with normal text.

| **Reference** | **Study Model/Type** | **Vitamin D supplementation** | | **Effects of Vitamin D** | |
| --- | --- | --- | --- | --- | --- |
|  |  | **Isoform - Dose - Duration - Administration** | **Control** | **Method/Type of experiment** | **Observation** |
| **In Vitro** | | | | | |
| Neveu et al. 1994 | Primary microglial cells from albino rats  (LPS or INFγ stimulated) | N/A | N/A | Immunocytochemistry | OX42+ microglia |
|  |  | [^3^H]-25(OH)D3 - 3 nM - 2 hours - Addition to medium | Untreated cells | HPLC | ↑ [^3^H]-1,25(OH)2D3 |
| Lefebvre d'Hellencourt et al. 2003 | EOC13 murine microglial cell line  (LPS or IFNγ stimulation) | 1,25(OH)2D3 - 1-10-100 nM - 12 hours - Addition to medium | Untreated cells | Cytokine bioassays | ↓ TNFα, ↓ IL-6 |
|  |  |  |  | Griess assay | ↓ NO production |
|  |  |  |  | RT-qPCR | *VDR+* |
|  |  |  |  | Northen blot | *= Tnfα, = lL-6, = iNos, = Tgf-β1* |
|  |  |  |  | ELISA | *↑* TGF-β1 (not significant) |
| Hur et al. 2014 | Primary microglia from Sprague-Dawley rats  (LPS stimulation) | 25(OH)D3 - 1-10-100 nM - 24 hours - Addition to medium | Untreated cells | Griess assay, siRNA | ↓ NO production (CYP27B1 and VDR dependent, siRNA) |
|  |  |  |  | MTT assay | = Cell viability |
|  |  |  |  | RT-qPCR | *Cyp27b* + (↑ upon LPS stimulation) |
|  | BV-2 murine microglial cell line  (LPS stimulation) | 25(OH)D3 - 1-10-100 nM - 24 hours - Addition to medium | Untreated cells | Griess assay, siRNA | ↓ NO production (CYP27B1 and VDR dependent, siRNA) |
|  |  |  |  | MTT assay | = Cell viability |
|  |  |  |  | RT-qPCR | *Cyp27b1*+ (↑ upon LPS stimulation) |
|  |  |  |  | Western blot | ↓ p-p38 (VDR dependent, siRNA) |
| Djukic et al. 2014 | Primary microglia from pups (p0-p2) of C57BL/6J mice (Vitamin D deficient diet)  TLR agonists stimulation | 25(OH)D3 - <100 IU/kg or 1500 IU/kg - 6 weeks - Addition to food, 6 weeks before mating | Primary microglial cell culture from pups (p0-p2) of C57BL/6J mice (Vitamin D deficient diet) | FACS | = F4/80 expression |
|  |  |  |  | Phagocytic assay | ↓ Phagocytosis of E. coli |
|  |  |  |  | Intracellular survival assay | ↓ Bacteria killed |
|  |  |  |  | Griess assay | = NO production |
|  |  |  |  | ELISA | ↓ IL-6, ↓TNFa, = CXCL1 |
| Smolders et al., 2013 | Primary microglia from human brain tissue  (TNFα + INFγ stimulation) | 1,25(OH)2D3 - 10 nM - 48 hours - Addition to medium | EtOH | RT-qPCR | ↓ *Cyp27b1,* ↑ *Cyp24a1,* |
| Kurtys et al. 2016 | BV-2 murine microglial cell line  (LPS stimulation) | 25(OH)D3 - 0.1-1 µg/ml - 24 hours - Addition to medium | EtOH | XTT assay | = Cell viability |
|  |  |  |  | Griess assay | ↓ NO production |
|  |  |  |  | ELISA | ↓ IL-6 |
| Boontanrart et al. 2016 | Primary microglia from SJL/J mice  (IFNγ or LPS stimulation or TMEV infection) | 25(OH)D3 or 1,25(OH)2D3 - 10 nM - 24 hours - Addition to medium | Untreated cells | RT-qPCR | *↑ Vdr, ↑ Cyp27b1, ↑ Cyp24a1, ↑ Socs3* (upon IFNγ or LPS stimulation or TMEV infection, 1,25(OH)2D3 further increases the levels)  *↑* *Il-10* (25(OH)D3 and 1,25(OH)2D3 in unstimulated cells)  ↓ *Il-6, ↑* *Il-10,* ↓ *Il-12,* ↓ *Tnfα,* ↓ *iNos,* ↓ *Mip-1α* (25(OH)D3 and 1,25(OH)2D3 in IFNγ stimulated cells) (IL-10 and SOCS3 dependent)  *↑* Inf*α, ↑* *Inf*β, ↓ *Il-6, ↑* *Il-10,* ↓ *Il-12,* ↓ *Tnfα,* ↓ *iNos,* ↓ *Mip-1α* (25(OH)D3 and 1,25(OH)2D3 in TMEV infected cells) (IL-10 and SOCS3 dependent) |
| Dulla et al. 2016 | BV-2 murine microglial cell line  (LPS stimulation and/or RXR antagonist HX630 treatment) | 1,25(OH)2D3 - 1 µM - 24 hours - Addition to medium | Untreated cells | RT-qPCR | ↓ *Il-1*β, ↓ *Il-6,* = *Tnfα,* ↓ *iNos* (also by HX630 alone, apart for IL-1 β) (combination with HX630 stronger effect for *iNos* and *Il-6*) |
|  |  |  |  | WST-8 Assay | *↑* Cell viability |
|  |  |  |  | Griess Assay | ↓ NO production (also by HX630 alone and even stronger effect in combination with HX630) |
|  |  |  |  | Western blot | ↓ iNOS (only when combined with HX630), ↓ pERK1/2 (in combination with HX630 stronger effect) |
|  |  |  |  | ELISA | ↓ Il-6 (only when combined with HX630) |
|  |  |  |  | Immunocytochemistry | ↓ NF-κB nuclear translocation (also by HX630 alone and in combination with HX630) (ERK dependent) |
| He et al. 2017 | Primary microglia from WT C57BL/6J, VDR^-/-^ and IL-10^-/-^ mice  (SEB infected) | 1,25(OH)2D3 - 10 nM - 48 hours - Addition to medium | Untreated cells | RT-qPCR, Western blot, shRNA and IL-10^-/-^ or VDR^-/-^ cells | ↑ VDR, ↓ TNFα (VDR and IL-10 dependent, shRNA and IL-10^-/-^ cells), ↑ IL-10 (VDR dependent, shRNA or VDR^-/-^ cells) |
|  |  |  |  | ChIP, Western blot, shRNA | ↓ LITAF binding to TNFα promotor (VDR dependent, shRNA) |
| Landel et al. 2018 | Primary microglia from Wistar rats | N/A | N/A | RT-qPCR | *Cyp27a1+*, *Cyp27b1*+, *Cyp24a1*+, *Pdia3*+, *Vdr*+ (low) |
|  |  | 1,25(OH)2D3 - 20 nM - 12 hours - Addition to medium | EtOH | RT-qPCR | *= Cyp27a1*, = *Cyp27b1*, ↑ *Vdr,* ↑ *Cyp24a1, = Pdia3* |
| Cui et al. 2019 | BV2 murine microglial cell line (Stimulated or not with Ang II) | 1,25(OH)2D3 - 1 µM - 30 minutes - Addition to medium, just before 24 hour exposure to Ang II | BV2 murine microglial cells (Stimulated or not with Ang II) | Western Blot | ↑ VDR (Ang II stimulation > without), = AT1, = ACE, ↑ ACE2 (Ang II stimulation > without), ↑ MasR (Ang II stimulation only)  With Ang II stimulation only: ↓ Nox2 (MasR dependent), = Nox4, ↓ p-p47phox (MasR dependent), ↓ p47phox (MasR dependent). ↓ NADPH oxidase activity (MasR dependent), ↓ ROS production (MasR dependent) |
|  |  |  |  | RT-qPCR | *↓ Il-1β (MasR dependent), ↓ Il-6 (MasR dependent), ↓ Tnfa (MasR dependent), ↓ Cd86 (MasR dependent), ↓ iNos (MasR dependent), ↑ Arg-1 (MasR dependent), ↑ CD206 (MasR dependent)* (from M1 to M2 phenotype) |
|  |  |  |  | Immunocytochemistry | With Ang II stimulation only: ↓ CD86 (MasR dependent), ↑ CD206 (MasR dependent) |
| Lee et al. 2020 | BV-2 murine microglial cell line  (LPS stimulation) | Conditioned medium from the N2a cell line treated with 1,25(OH)2D3 - 10-100-1000 nM - 24 hours - Addition to medium (8 hours) | EtOH | RT-qPCR | *↓ MhcII, ↓ iNos, ↑ Hmox1, ↑ Arg1* |
|  |  |  |  | ELISA | ↓ IL-6 (IL-34 dependent) |
|  | Primary microglia from P1 mice  (LPS stimulation) | Conditioned medium from primary neurons treated with 1,25(OH)2D3 - 10-100-1000 nM - 24 hours - Addition to medium (24 hours) | EtOH | ELISA | ↓ IL-6, *↓* IL-1β, = TNFα |
| Clarke et al. 2020 | Primary human microglia from patients undergoing surgery for intractable epilepsy | 1,25(OH)2D3 - 100 nM - 6 days - Addition to medium | Vehicle (not mentioned) | Bulk RNAseq (PCA, ORA) | Microglia cluster along the first principal component based on their cellular phenotype (MG_0_ and MG_GMcsf_) and based on treatment with calcitriol (PCA)  Differentially expressed genes in “inflammatory response” and “cytokine production/secretion” (ORA) |
|  |  |  |  | RT-qPCR | ↓ *MerTK* (MG_0_ and MG_GMcsf_) |
|  |  |  |  | FACS | ↓ MerTK (MG_0_ only) |
| Alessio et al. 2021 | Primary microglia from pups (p2-p4) of C57BL/6J mice  (Vitamin D deficient diet) | Cholecalciferol - <5 IU/kg or 1500 IU/kg - 3 weeks - Addition to food (3 weeks before mating, 3 weeks till pups delivery, 1 week after pup delivery) | Primary microglial cultures from pups (P2-4) of C57BL/6J mice  (Vitamin D suficient diet) | Immunocytochemistry | ↑ Total cell number, ↑ Activated cells (↑ Soma size, ↑ Process lengths), ↑ Iba1+ cells |
|  |  |  |  | FACS | ↑ Ki67+ cells, ↑ ATM+ cells |
|  |  |  |  | Cell Counting Kit-8 (CCK-8) | = Cell viability |
|  |  |  |  | DCFH-DA assay | ↑ Intracellular ROS production |
|  |  |  |  | D-ROM Test | = Media ROS levels |
|  |  |  |  | Nexin V assay | = Annexin V+ cells, = 7AAD+ cells (= Apoptosis) |
|  |  |  |  | In Situ Senescence-Associated β-Galactosidase Assay | ↑ β-Gal+ cells |
|  | Primary microglia from pups (p2-p4) of C57BL/6J mice  (Vitamin D deficient diet) | 25(OH)D3 or 1,25(OH)2D3 - 100 nM - 24 hours - Addition to medium | DMSO 0.001% or 100 nM PEA | FACS | ↓ Ki67+ cells (VitD deficient derived cells only), = Cell cycle state, = ATM+ cells, |
|  |  |  |  | Cell Counting Kit-8 (CCK-8) | = Cell viability |
|  |  |  |  | DCFH-DA assay | ↑ Intracellular ROS production |
|  |  |  |  | D-ROM Test | = Media ROS levels |
|  |  |  |  | Nexin V Assay | = Annexin V+ cells, = 7AAD+ cells (= Apoptosis) |
|  |  |  |  | In Situ Senescence-Associated β-Galactosidase Assay | ↓ β-Gal+ cells |
| **In Vivo** | | | | | |
| Nataf et al. 1996 | Lewis rats  GPSC induced chronic relapsing EAE | 1,25(OH)2D3 - 5 µg/kg and 1 µg/kg - 23 days - i.p., starting with 5 µg/kg on d11 and d13 post immunisation and continuing with 1 µg/kg on d19, d21, d23 | Propylene glycol and disodium phosphate | Clinical score | ↓ Disease incidence, ↓ Clinal score at d23 |
|  |  |  |  | Immunohistochemistry | = MD-1+ microglia/macrophages, ↓ OX42+ microglia/macrophages in the anterior brain (= in the rest of the CNS), ↓ CD4+ T-cells/microglia/macrophages in all CNS parts |
| Garcion et al. 1997 | Lewis rats  GPSC induced chronic relapsing EAE | 1,25(OH)2D3 - 5 µg/kg and 1 µg/kg - 23 days - i.p., starting with 5 µg/kg on d11 and d13 post immunisation and continuing with 1 µg/kg on d19, d21, d23 | Propylene glycol and disodium phosphate | Immunohistochemistry | ↓ OX42+ microglia/macrophages in the anterior brain (= in the rest of the CNS) |
|  |  |  |  | In-situ hybridisation | ↓ OX42+iNOS+ microglia/macrophage in the cerebellum, brainstem and spinal cord ( = in the anterior brain) |
| Garcion et al. 1998 | Lewis rats  Hippocampal lesion and stereotactic LPS injection | 1,25(OH)2D3 - 1000nM - 5-15-24-48-72 hours - Simultaneous stereotactic injection of LPS and VitD in the hippocampus | PBS | Immunohistochemistry | ↑ OX42+ microglia/macrophages (72h only) |
|  |  |  |  | TUNEL assay | = Cell death |
|  |  |  |  | In Situ Hybridisation | ↓ OX42+iNOS+ microglia/macrophages (at 15-24h) |
| Nashold et al. 2000 | B10.PL(73NS)/Sn mice  MBP EAE | 1,25(OH)2D3 - 100 ng (injection) + 50 ng (diet) - 72 hours - i.p., every day, starting at moderate EAE severity + Additional food supply every day | Soybean oil (injection), 1,25(OH)2D3 devoid diet | Immunohistochemistry | ↑ Mac-1+ macrophages/microglia |
| Garcion et al. 2003 | Lewis rats  GPSC induced chronic relapsing EAE | 1,25(OH)2D3 - 5 µg/kg and 1 µg/kg - 23 days - i.p., starting with 5 µg/kg on d11 and d13 post immunisation and continuing with 1 µg/kg on d19, d21, d23 | Propylene glycol and disodium phosphate | Clinical score | ↓ Incidence of score 4 first paralytic attack, earlier second paralytic attack  (Similar kinetics in Vitamin D3 deprived animals) |
|  |  |  |  | Immunohistochemistry | ↓ OX42+ cells, ↓ MHCII+ cells, ↓ CD4+ cells, ↓ iNOS+ cells |
|  |  |  |  | In situ hybridization | *↓ iNos+, = Tgf-β1* |
| Kim et al. 2006 | Sprague-Dawley rat  6-OHDA model of Parkinson’s disease | 1,25(OH)2D3 - 1 μg/ml at 1 ml/kg/day - 7 days - i.p., every day, starting 7 days before 6-OHDA injection | Ethanol 0.1% | Immunohistochemistry | ↑ Tyrosine hydroxylase+ cells, ↓ CD11b+ activated microglia |
|  | C57BL/6J mice  MPTP model of Parkinson’s disease | 1,25(OH)2D3 - 1 μg/ml at 1 ml/kg/day - 7 days - i.p., every day, starting 7 days before MPTP injection | Ethanol 0.1% | Immunohistochemistry | ↓ CD11b+ activated microglia |
|  |  |  |  | RT-qPCR | *↓ Tnfα, ↓ Infγ* |
| Wergeland et al. 2011 | C57BL/6J mice  Cuprizone Model | Not mentioned (Vitamin D3) - 50, 500, 6200, 12’500 IU/kg - 10 weeks - Addition to food, every day, starting 2 weeks before cuprizone model | Cuprizone untreated C57BL/6J mice | Immunohistochemistry | ↓ Number of Mac-3+ activated microglia/macrophages |
| Nystad et al. 2014 | C57BL/6J mice  Cuprizone Model | 1,25(OH)2D3 - 0.2 ug/mouse - 4 weeks - i.p., twice weekly, starting 5 weeks after cuprizone treatment | Vehicle (not mentioned) | Immunohistochemistry | ↓ Mac-3+ activated microglia/macrophages (only after 1-week remyelination) |
| Calvello et al. 2017 | C57BL/6N mice  MPTP model of Parkinson’s disease | 1,25(OH)2D3 - 1 μg/kg - 10 days - Intragastric gavage, starting 3 days before MPTP injection | Saline solution | Immunohistochemistry | ↓ Tyrosine hydroxylase+ cells, ↓ Iba1+ cells  On Iba-1+ cells: ↑ CD206, ↑ CD163, ↑ CD204 |
|  |  |  |  | Western blot | ↓ Tyrosine hydroxylase, ↓ Iba1, ↓ TLR-4, ↓ iNOS, ↑ CD206, ↑ CD163, ↑ CD204 |
|  |  |  |  | RT-qPCR | *↓ Tnfα, ↓ Il-1β, ↑ Il-10, ↑ Tgfβ, ↑ lL-4* |
| Evans et al. 2018 | C57BL/6J mice  SAH model | 1,25(OH)2D3 - 100 ng/kg/day - 5 days - i.p., every day, starting 5 days prior SAH | Sterile water, propylene  glycol and ethanol (5:4:1) | Immunohistochemistry | ↑ M2 microglia/macrophages (CD206+ cells) |
| Cui et al. 2019 | Wistar–Kyoto (WKY) rats and Spontaneous Hypertensive (SHR) rats | 1,25(OH)2D3 - 100 ng/kg - 6 weeks - Oral gavage, every day | Saline solution | Body weighting | = Body weight |
|  |  |  |  | Immunohistochemistry, Western blot | ↑ VDR (WKY>SHR)  In SHR rats brain only: ↓ Apoptosis, ↑ Neuron viability, = ACE, ↓ Ang II, = AT1 (VDR co-localized), ↑ ACE2, ↑ Ang (1–7), ↑ MasR (VDR co-localized), ↓ Nox2, ↓ p-p47phox, = p47phox, ↓ Nox4, ↓ total Nox activity, ↓ DHE, ↓ MDA, ↑ SOD activity, ↑ CAT activity |
| de Oliveira et al. 2020 | C57BL/6J mice  MOG_35-55_ EAE | 1,25(OH)2D3 - 5 µg/kg - 16 days - i.p, every two days, starting 24 hours after immunization | Saline solution | FACS | ↓ CD45^High^CD11b^+^ cells (activated microglia), = CD45^Low^CD11b^+^ cells (resting microglia), ↓ MHC-II on activated and resting microglia, = PD-L1 on activated and resting microglia |
| Spanier et al. 2020 | C57BL/6 (B6) mice  (Vitamin D deficient diet) | 1,25(OH)2D3 - 0 or 1 μg/day - 7-8 weeks - Food supply, starting 3-4 weeks before EAE induction | C57BL/6 (B6) mice  (Vitamin D sufficient diet) | Clinical score analysis | ↑ Disease incidence, ↑ Peak disease score, ↑ Cumulative disease index |
|  |  |  |  | FACS | ↓ CTLA-4+ Conventional T cells and Tregs, ↓ CTLA-4 MFI on Conventional T cells and Tregs (trend) |
|  | C57BL/6 (B6) mice lacking exon 8 of the Cyp27b1 gene in myeloid lineage cells (M-Cyp27b1^0^)  (Vitamin D deficient diet)  MOG_35-55_ EAE | 1,25(OH)2D3 - 0 or 1 μg/day - 7-8 weeks - Food supply, starting 3-4 weeks before EAE induction | C57BL/6 (B6) mice lacking exon 8 of the Cyp27b1 gene in myeloid lineage cells (M-Cyp27b1^0^)  (Vitamin D sufficient diet) | Clinical score analysis | = Disease incidence, = Peak disease score, = Cumulative disease index |
|  |  |  |  | FACS | = CTLA-4+ Conventional T cells and Tregs, = CTLA-4 MFI on Conventional T cells and Tregs (trend) |
| Alessio et al. 2021 | C57BL/6J mice (Vitamin D deficient diet) | Cholecalciferol - <5 IU/kg or 1500 IU/kg - 7 weeks - Food supply (3 weeks before mating, 3 weeks till pups delivery, 1 week after pup delivery) | C57BL/6J mice (Vitamin D sufficient diet) | Quantitative chemiluminescent microparticle immunoassay | ↓ Serum 25(OH)D3 levels |
|  |  |  |  | D-ROM Test | = Serum ROS levels |
|  |  |  |  | Simplified Up-Down method | ↓ Tactile withdrawal threshold |
|  |  |  |  | Immunohistochemistry | = Number of Iba-1+ cells, ↑ Activated Iba-1+ cells (more prominent in females), ↑ Dystrophic Iba-1+ cells (more prominent in females) |
| **MS patients / derived samples** | | | | | |
| Smolders et al. 2013 | Postmortem brain tissue of MS and control patients (NAWM and active lesions) | N/A | N/A | Immunohistochemistry | HLA+VDR+ macrophages/microglia (both MS and control patients, in both NAWM and active lesions), HLA+CYP24A1- macrophages/microglia (both MS and control patients, in both NAWM and active lesions) |
|  |  |  |  | RT-qPCR | *↑ Vdr, ↑ Cyp27b1* (in chronic active lesions vs NAWM) |

**Supplementary Table 7. The effects of vitamin D on microglia.**

The effects of Vitamin D are given as ↑ increased; ↓ decreased; = not affected. Changes in protein and mRNA expression levels are indicated respectively with normal and *italic* text. Combined data on protein and mRNA are written with normal text.
